# Riluzole shifts glial responses to protect synapses and memory in Aβ oligomer–treated rats

**DOI:** 10.1186/s12974-026-03972-3

**Published:** 2026-08-25

**Authors:** Min-Kaung-Wint-Mon, Hiroyuki Kida, Ryoichi Kimura, Dai Mitsushima

**Affiliations:** 1 Department of Physiology, Yamaguchi University Graduate School of Medicine, Yamaguchi, 755-8505 Japan; 2 Center for Liberal Arts and Sciences, Sanyo-Onoda City University, Sanyo- Onoda, Yamaguchi, 756-0884 Japan; 3 The Research Institute for Time Studies, Yamaguchi University, Yamaguchi, 753-8511 Japan

**Keywords:** Aβ_1-42_ oligomers, Contextual memory, Astrocytes, Microglia, Synaptic loss, Riluzole

## Abstract

**Background:**

Soluble Aβ_1-42_ (amyloid beta) oligomers are potent neurotoxins that disrupt synaptic function, alter glial responses, and lead to memory impairment in Alzheimer’s disease (AD). Riluzole, a glutamate modulator approved for amyotrophic lateral sclerosis, reduces neuronal hyperexcitability, yet its in vivo effects on Aβ oligomer–induced cognitive dysfunction and glial alterations remain incompletely understood. Here, we investigated whether riluzole ameliorates Aβ_1-42_ oligomer–induced memory impairment and associated hippocampal pathology.

**Methods:**

Aβ_1-42_ oligomers were bilaterally microinjected into the dorsal CA1 region of freely moving male rats, followed by daily riluzole administration for 7 days. Then, the animals underwent a battery of behavioural tests before being sacrificed for immuno-histochemical studies. Glial engulfment of Aβ and synapse was visualized through multiplex immunohistochemistry combined with super-resolution microscopy and three-dimensional (3D) reconstruction.

**Results:**

Behavioral analyses revealed that riluzole significantly improved hippocampus-dependent memory, including contextual learning and spatial working memory, without affecting locomotor activity, anxiety-like behavior, or pain sensitivity. At the cellular level, riluzole reduced neuronal Aβ accumulation which was strongly associated with improved learning performance across individual animals. It enhanced robust Aβ internalization and lysosomal processing within astrocytes and microglia. Though Aβ-induced glial recruitment and activation persisted, riluzole enhanced morphological remodeling and shifted glial cells towards neuroprotective phenotype. Riluzole also attenuated Aβ-induced neuronal apoptosis, dendritic degeneration, and dendritic spine loss across the dorsal CA1 sublayers, and limited complement-mediated synaptic elimination by microglia.

**Conclusion:**

By enhancing glial Aβ clearance and preserving synaptic integrity, riluzole effectively counteracts Aβ-driven cognitive decline. Together, these findings highlight coordinated glial remodeling as a physiologically relevant mechanism at a therapeutically actionable stage of AD.

**Supplementary Information:**

The online version contains supplementary material available at https://doi.org/10.1186/s12974-026-03972-3.

## Introduction

The aggregation and accumulation of the β-amyloid (Aβ) peptide are central features of Alzheimer’s disease (AD) pathogenesis [1]. While early formulations of the amyloid hypothesis emphasized the role of insoluble plaques, subsequent work identified soluble Aβ_1-42_ oligomers as key neurotoxic species that initiate synaptic dysfunction and cognitive decline [1, 2]. Consistent with this view, intracerebral administration of natural or synthetic Aβ_1-42_ oligomers induces synaptic loss, astrogliosis, neuronal death, and memory impairment in rodents [3, 4]. These findings position soluble Aβ_1-42_ oligomers as a principal pathogenic driver of AD-related cognitive dysfunction.

A growing body of evidence indicates that soluble Aβ oligomers profoundly disrupt glutamatergic signaling, leading to neuronal hyperexcitability and network instability [5–7]. Such glutamatergic dysregulation impairs long-term potentiation (LTP) and disturbs the excitatory/inhibitory (E/I) balance, ultimately compromising learning and memory [8, 9]. In parallel, excessive extracellular glutamate and overactivation of glutamatergic receptors promote Ca²⁺ overload, synaptic damage, and neuronal death [10–12]. Together, these observations underscore glutamatergic dysfunction as a critical and therapeutically relevant mechanism in Aβ-induced pathology.

Neuronal hyperexcitability is closely linked to Aβ deposition [13] and cognitive impairment in AD [5, 14], potentially through dysregulation of voltage-gated sodium channel (Nav) and persistent sodium current (I_*NaP*_) [15]. Consistently, suppression of Nav1.6 expression or activity reduces amyloid burden and improves cognitive performance [16, 17]. These findings suggest targeting Nav1.6-driven hippocampal hyperexcitability may represent a new potential therapeutic strategy for AD.

Riluzole (2-amino-6-(trifluoromethoxy)benzothiazole), a FDA-approved treatment for amyotrophic lateral sclerosis (ALS), is a glutamate modulator that reduces presynaptic glutamate release and enhances glutamate reuptake [18, 19]. Through these actions, riluzole stabilizes excitatory network activity and improves synaptic plasticity, including LTP [9, 20]. In addition to its effects on neuronal excitability [21], riluzole has been shown to reverse age-associated transcriptional changes, synaptic alterations, and memory decline [22, 23], highlighting its broad neuroprotective potential [24, 25]. These properties suggest riluzole may be well suited to counteract Aβ-driven pathology in AD.

We previously demonstrated that riluzole, a I_*NaP*_ blocker, suppresses Aβ_1-42_ oligomer–induced neuronal hyperexcitability in vitro [21]. However, whether riluzole can ameliorate Aβ oligomer–induced cognitive dysfunction in vivo, and how it affects the cellular mechanisms remain incompletely understood. In the present study, we found that riluzole rescued Aβ_1-42_ oligomer-induced memory impairment by modulating glial responses to Aβ pathology. Specifically, riluzole preserved synaptic integrity and cognitive function by enhancing glial Aβ clearance, limiting synaptic pruning as well as promoting functional remodeling.

## Materials and methods

### Animals

Male Sprague–Dawley rats (4 weeks old; Chiyoda Kaihatsu, Japan) were housed under standard laboratory conditions (23 ± 1 °C, 12 h light/dark cycle) with ad libitum access to food and water. Only males were used in this study to reduce variability associated with estrous cycle–dependent hormonal fluctuations. Estrogen has been reported to modulate Aβ-induced neurotoxicity and cognitive impairment [26]; therefore, restricting the experimental cohort to males allowed more precise assessment of the effects of Aβ_1−42_ oligomers and riluzole.

A total of 35 rats were used, and all procedures were conducted in accordance with institutional guidelines to minimize animal number and suffering.

### Experimental design

**Fig. 1:**
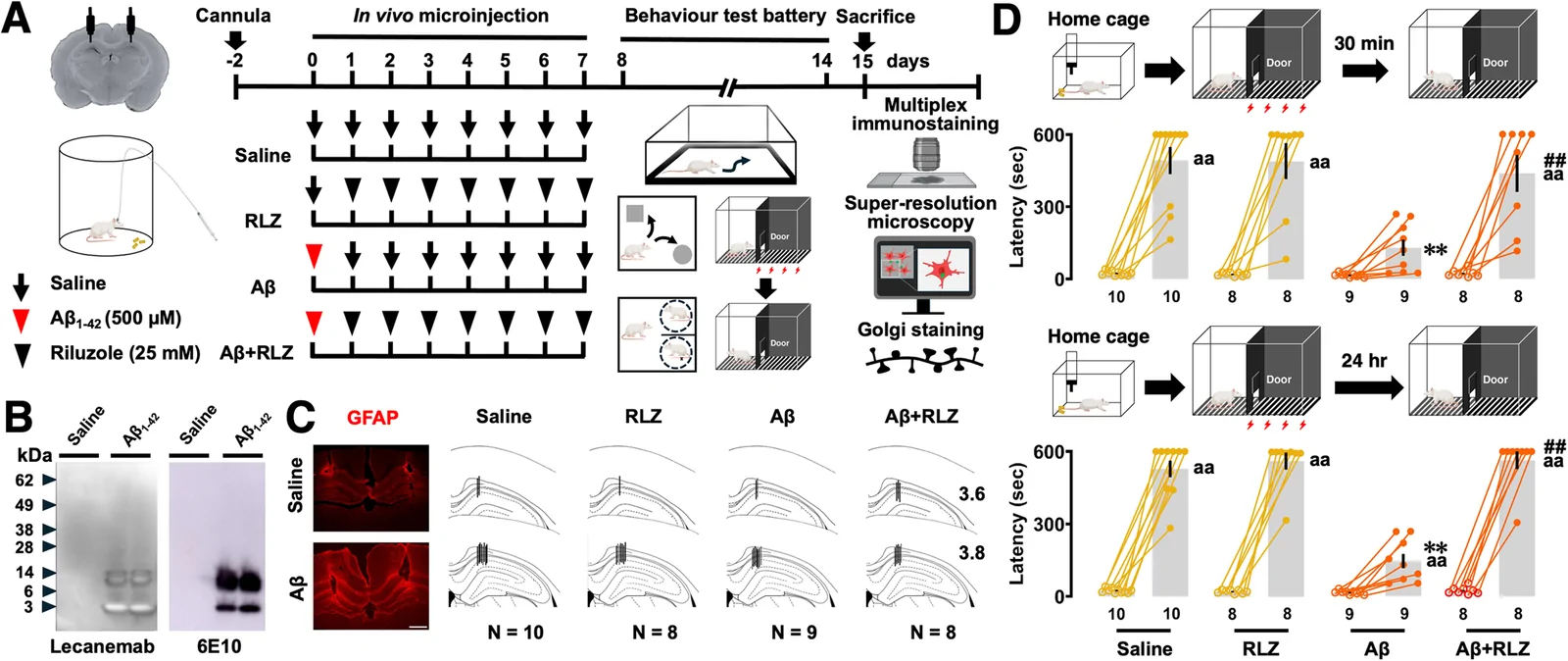
Riluzole attenuates Aβ_1-42_ oligomer–induced contextual memory impairment in the inhibitory avoidance (IA) task. **A** Experimental timeline and schematic of in vivo microinjection. A guide cannula was implanted above the dorsal CA1 region, and rats received bilateral microinjection of Aβ_1-42_ oligomers followed by daily riluzole administration for 7 days. Then, the animals underwent a battery of behavioural tests before being sacrificed for staining. Glial phagocytosis of Aβ and synapse was visualized by multiplex immunohistochemistry, super-resolution microscopy and 3D reconstruction. **B** Representative immunoblots of Aβ_1-42_ oligomer preparation separated by 4–20% gradient SDS-PAGE and probed with anti-Aβ antibodies (lecanemab and 6E10). **C** Representative GFAP immunofluorescence images showing astrocytic responses after Aβ_1-42_ oligomer injection (left) and schematic reconstructions of microinjection sites in the dorsal hippocampus (right). Numbers indicate the distance (mm) posterior to bregma. Scale bar, 1000 µm. **D** Contextual learning assessed by the IA task. Schematic illustrations of the task were shown above the plots. Rats received a foot shock (1.6 mA, 2 s) upon entry into the dark compartment during training. Latency to enter the dark compartment was measured 30 min (top) and 24 hr (bottom) after training. Open and closed circles indicate training and retrieval data, respectively. Data were shown as mean ± SEM; sample sizes were indicated below each group. ^aa^P < 0.01 vs training; **P < 0.01 vs saline; ^##^P < 0.01 vs Aβ. Illustrations were created using BioRender

To examine the effects of riluzole on Aβ_1-42_oligomer–induced memory impairment, Aβ oligomers were bilaterally microinjected into the dorsal CA1 region, followed by daily riluzole administration for 7 days. Then, the rats underwent a battery of behavioural test before being sacrificed for staining. Glial phagocytosis of Aβ and synapse was visualized by multiplex immunohistochemistry combined with super-resolution microscopy and 3D reconstruction (Fig. 1A).

### Preparation of Aβ_1−42_ oligomers

Synthetic Aβ_1−42_ peptide was prepared according to a previously established oligomerization protocol [27], with minor modifications. Briefly, lyophilized Aβ_1−42_ peptide was dissolved in hexafluoro-2-propanol (HFIP) to disaggregate pre-existing assemblies, dried under vacuum, and reconstituted in dimethyl sulfoxide (DMSO). The peptide solution was then diluted in phosphate-buffered saline (PBS) to obtain an oligomer-enriched preparation and incubated at 4 °C for 24 h. Aliquots were stored at − 40 °C until use and diluted in 0.9% NaCl immediately before microinjection. The final DMSO concentration was 0.4%.

This preparation is referred to as ‘Aβ oligomers’ throughout the manuscript, and all experiments were performed using the same preparation batch.

### Western blotting

The oligomeric composition of Aβ preparation was verified by SDS–PAGE under non-denaturing and non-reducing conditions. Samples were prepared by mixing Aβ_1−42_ peptide (100 µM) with 2× Laemmli sample buffer at a 1:1 ratio. Aliquots (10 µl) were separated on 4–20% gradient SDS–PAGE gels (Mini-PROTEAN TGX, Bio-Rad) and transferred onto PVDF membranes (0.45 μm). Membranes were blocked with 5% skim milk in TBS-T and incubated overnight at 4 °C with lecanemab (1:5000; A3112, Selleck Biotechnology) or 6E10 (1:5000; 803004, BioLegend). After washing, membranes were incubated with HRP-conjugated anti-human or anti-mouse secondary antibodies (1:5000) for 1 h at room temperature. Immunoreactive bands were visualized using enhanced chemiluminescence and imaged with an Amersham Imager 680.

Immunoblotting with lecanemab detected a mixture of low–molecular weight species (monomers to tetramers) and higher-order oligomers, whereas immunodetection with 6E10 predominantly revealed low–molecular weight Aβ species (Fig. 1B).

### Stereotaxic surgery and microinjection

Rats were anesthetized with ketamine (100 mg/kg) and xylazine (10 mg/kg) and placed in a stereotaxic frame. Stainless-steel guide cannula was implanted bilaterally above the dorsal hippocampal CA1 region (AP − 3.0 mm, ML ± 2.0 mm, DV − 1.0 mm from dura).

On the experimental day, the stylet was replaced with a 1-mm longer injector (outer diameter = 0.31 mm) through a fine, flexible silicone tubing (outer diameter = 0.5 mm, Kaneka Medix Co. Osaka, Japan). In vivo microinjection was performed under freely moving conditions. Aβ_1−42_ oligomers (500 µM, 1 µL per side) were bilaterally delivered at a rate of 1 µL/5 min on Day 0. Riluzole (25 mM, 1 µL per side; A2423, TCI, Tokyo, Japan) was then administered once daily via the same route for the subsequent 7 days.

Saline (1 µL per side) was used as vehicle for both Aβ oligomers and riluzole (Fig. 1A). Injectors were left in place for 5 min after infusion to prevent reflux. Cannula placement was verified histologically. In addition, GFAP immunoreactivity was increased in the target region after Aβ oligomer injection, indicating astrocytic recruitment in response to Aβ (Fig. 1C).

### Behavioral test battery

After riluzole treatment, all rats underwent a behavioral test battery to assess hippocampus-dependent memory and basic physiological functions (Figure S1). Animals were habituated to the testing environment, and behavior was recorded by video. Apparatuses were cleaned between trials, and a 30 min inter-test interval was maintained [21, 28].

### Open field test

Locomotor activity and anxiety-related behavior were assessed by recording total distance traveled, center time, center entries, and rearing during a 5-min session [21].

### Object-based memory tasks

Exploration was defined as directing the nose toward an object within 2 cm or touching it with the forelimbs for ≥ 1 s; sitting or leaning against objects was excluded.

- Object recognition: One familiar object was replaced with a novel object during the test phase.
- Object location: One familiar object was moved to a new location.
- Object-in-place: Two of four objects were exchanged.
- Object recency: One object from the first sampling phase and one from the second were presented.

Memory performance was quantified as the ratio of exploration time for the novel object or location to total exploration time [28, 29]. We measured the time spent interacting with the objects after 5-min, 30-min and 24-h intervals.

### Y-maze task

Working memory was assessed by spontaneous alternation behavior during a 5-min session. After 24 h, the rats were placed in one of the arms again, and their behavior was recorded for 5 min. Alternation percentage was calculated as (actual alternations / possible alternations) × 100 [21].

### Social behavior and memory

Social interaction time was measured during free interaction with an unfamiliar partner. Social preference and recognition were assessed using a two-choice task, and performance was expressed as the ratio of interaction time with the novel social target to total interaction time [21].

### Inhibitory Avoidance (IA) task

Contextual learning was assessed by measuring latency to enter the shock-associated compartment during test sessions conducted 30 min and 24 h after training [21, 30].

### Fear conditioning test

Rats received three-foot shocks (0.8 mA, 2 s) during training. Freezing behavior was assessed 1 h and 24 h later [21]. Freezing was defined as the cessation of all behaviors except respiration for at least 1 s.

### Light/dark test

Anxiety-like behavior was evaluated by measuring time spent in the lit compartment and rearing frequency [21].

### Flinch-jump test

Pain sensitivity was evaluated by determining flinch, jump, and vocalization thresholds in response to increasing foot shock intensities [21].

### Brain section and immunohistochemistry

Free-floating immunohistochemistry was performed as previously described [21]. Briefly, brains were perfused, fixed, cryoprotected, and sectioned (30 μm). Sections were incubated with primary and secondary antibodies (Table S1), mounted, and imaged under identical conditions.

### Multiplex fluorescent immunohistochemistry

For visualization of glial phagocytosis of Aβ and synapse, multiplex immunostaining was performed sequentially with secondary antibodies conjugated with Alexa Fluor 488, 594 and 647 and counterstained with DAPI.

### Image acquisition and quantification

Images were acquired under identical conditions an all-in-one fluorescence microscope (BZ-X810, KEYENCE Co., Osaka, Japan). Cell density was calculated as cells/mm² and fluorescence intensity was quantified using ImageJ (National Institutes of Health, https://imagej.net/ij/). 2–3 sections per animal (4–5 per group) were analyzed by an experimenter blind to treatment.

### Morphological analysis of astrocytes and microglia

Cell morphology was reconstructed from z-stack images. Soma area, cell area, territory area, process length, branch number and Sholl intersections were quantified using Fiji/ImageJ software (https://imagej.net/software/fiji/).

### Classification of microglia

Microglia were classified as ramified, hypertrophic, dystrophic, amoeboid, or rod-shaped based on established morphological criteria [31].

### Analysis of synapses

Z-stack images of 5 μm thickness (0.5 μm step-size) were captured with 40×/0.95 NA objective lens (3× digital zoom) at 960 × 720 pixel under identical conditions. The images were converted into 3D images and visualized by 3D viewer plug-in. For quantification of synaptic colocalization, 2–3 ROIs (region of interest) from SR (stratum radiatum) were randomly selected. To analyze colocalization, the image was threshold-adjusted and puncta (0.5–5 µm^2^) were measured using ImageJ Synbot plugin and expressed as puncta per 100 µm^2^. Internalized synaptic markers within microglia were quantified manually.

### Super-resolution microscopy and 3D reconstruction

Representative confocal images were captured by Leica Stellaris 8 STED microscope and acquired using Leica application suite X (LASX) software. ROI was imaged using a 40× oil-immersion objective lens at 1024 × 1024 pixel with ×1 zoom, 1.039 μm optical section thickness and 1.0 AU pinhole. Z-stacks were acquired with a 0.5 μm step size to enable accurate 3D reconstruction, covering a total depth of 15–24 μm depending on the structure of interest. The voxel size was 0.284 × 0.284 × 1 µm^3^. The field of view was 290.63 × 290.63 µm^2^ and each stack contained 31–49 planes. Laser power was kept below 10% to minimize photobleaching while preserving adequate signal-to-noise ratio, and detector gain was individually optimized for each channel to avoid saturation. Fluorescence signals were detected using Stellaris Power HyD detectors with pre-defined spectral windows, and multi-channel imaging was performed through line sequential acquisition to minimize crosstalk between fluorophores while allowing real-time acquisition.

Z-stacks were deconvolved, converted into IMS files and processed using Imaris (v.10.0.1; Bitplane) software.

### Visualization of glial engulfment

Imaris 3D surface rendering was performed as described previously [32, 33]. First, a surface was created for the cell (GFAP or Iba1) channel. The lysosome (Cathepsin D or CD68) channel was then masked to the cell surface to obtain the lysosome within the cell which was then surface rendered. A mask was applied for Aβ or PSD95 channel to obtain amyloid or synapse inside the cell. Surfaces were created for the masked Aβ or PSD95 channel. Colocalization of Aβ or PSD95 with CD68 within microglia or Aβ/CathD with astrocytes was defined as glial phagocytosis. To visualize engulfed and unengulfed Aβ, Aβ channel was masked outside and inside the surface (Figure S2).

### Visualization of synapse colocalization

First, a surface was rendered for both pre-and post-synaptic markers. A new surface was created with the shortest distance to nearest neighboring surface filter set below 0.005 μm.

#### Golgi-Cox staining and spine analysis

Golgi-Cox staining was performed as previously described [34]. Spine density and morphology were analyzed along 20-µm dendritic segments across the dorsal CA1 sublayers. Classification of spines was performed by measuring head width, neck width and neck length. Spines were categorized as ‘thin’ if the head width exceeded the neck width and if the neck length was greater than its width. ‘Mushroom’ spines were defined by a prominent head in which the head width was greater than the neck width which was also greater than its length. Spines were defined as ‘stubby’ if the neck width was similar to the spine length [35, 36].

### Statistical analysis

Data from slices, ROIs or dendritic segments were averaged per animal and presented as mean ± SEM. Normality was assessed using the Shapiro–Wilk test. Behavioral and cellular data were analyzed using two-way ANOVA with Aβ and riluzole as factors, followed by Bonferroni post hoc tests. Recognition memory tasks were analyzed using paired t-tests comparing novel versus familiar targets. The association between contextual learning and neuronal Aβ burden was assessed by Pearson’s correlation analysis. Statistical significance was set at *p* < 0.05.

## Results

### Effect of riluzole on Aβ_1−42_ oligomer–induced contextual memory impairment

To examine the effects of riluzole on Aβ_1−42_ oligomer–induced memory impairment, contextual learning was assessed using the inhibitory avoidance (IA) task (Fig. 1D). Thirty minutes after training, a three-way ANOVA revealed significant main effects of training (*F*₁,₆₂ = 147.503, *P* < 0.0001), Aβ (*F*_1,62_ = 12.076, *P* = 0.0009), and riluzole (*F*_1,62_ = 6.675, *P* = 0.0121), as well as a significant interaction among these factors (*F*_1,62_ = 6.220, *P* = 0.0153). Post hoc analyses showed that training increased step-through latency in the saline, RLZ, and Aβ + RLZ groups, whereas this increase was not observed in the Aβ group.

When retention was reassessed 24 h after training, a three-way ANOVA again revealed significant main effects of training (*F*_1,62_ = 626.622, *P* < 0.0001), Aβ (*F*_1,62_ = 31.983, *P* < 0.0001), and riluzole (*F*_1,62_ = 44.963, *P* < 0.0001), together with a significant interaction (*F*_1,62_ = 29.094, *P* < 0.0001). Although step-through latency increased across all groups at 24 h, Aβ-treated rats exhibited reduced latency relative to controls, whereas riluzole treatment increased latency in Aβ-injected rats. Together, these results indicate that riluzole attenuates Aβ_1−42_ oligomer–induced deficits in contextual memory.

### Effect of riluzole on sensory, motor, and emotional functions

Because performance in hippocampus-dependent memory tasks can be influenced by basic physiological and emotional factors, we assessed locomotor activity, anxiety-like behavior, pain sensitivity, object exploration, and social behavior.

In the open-field test, there were no significant group differences in total distance traveled (Aβ: *F*_1,31_ = 0.011, *P* = 0.9160), time spent in the center (Aβ: *F*_1,31_ = 2.459, *P* = 0.1270), latency to enter the center (Aβ: *F*_1,31_ = 0.250, *P* = 0.6206), frequency of center entries (Aβ: *F*_1,31_ = 0.204, *P* = 0.6547), or rearing activity (Aβ: *F*_1,31_ = 1.198, *P* = 0.2822), indicating that neither locomotor activity nor exploratory behavior was altered across groups (Figure S3A). To determine whether motivation influenced recognition performance, object exploration and social behaviors were examined. Total object exploration time did not differ among groups (Figure S3B; Aβ: *F*_1,31_ = 0.210, *P* = 0.6501). Similarly, no group differences were observed in total social interaction time (Aβ: *F*_1,31_ = 0.061, *P* = 0.8072) or social preference (Aβ: *F*_1,31_ = 0.661, *P* = 0.4225), indicating comparable motivation to explore objects and engage in social interactions (Figure S3C).

Consistent with these findings, performance in the light/dark test revealed no significant differences in time spent in the light compartment (Aβ: *F*_1,31_ = 0.305, *P* = 0.5846), latency to enter the light compartment (Aβ: *F*_1,31_ = 0.216, *P* = 0.6457), frequency of light compartment entries (Aβ: *F*_1,31_ = 2.165, *P* = 0.1512), or rearing activity (Aβ: *F*_1,31_ = 0.018, *P* = 0.8939), indicating no detectable changes in anxiety-like behavior (Figure S3D). Finally, pain sensitivity assessed by the flinch–jump test was not altered, as there were no significant differences in flinch (Aβ: *F*_1,31_ = 3.795, *P* = 0.0605), vocalization (Aβ: *F*_1,31_ = 0.378, *P* = 0.5432), or jump thresholds (Aβ: *F*_1,31_ = 0.100, *P* = 0.7542) (Figure S3E).

Together, these results indicate that riluzole does not affect sensory, motor, or emotional functions, supporting the conclusion that the observed improvements in hippocampus-dependent memory are not attributable to nonspecific behavioral confounds.

### Effect of riluzole on Aβ_1-42_ oligomer–induced impairment in hippocampus-dependent memory tasks

We next examined whether riluzole rescues deficits in multiple hippocampus-dependent memory domains using a battery of recognition, associative, social, and contextual tasks.

**Fig. 2:**
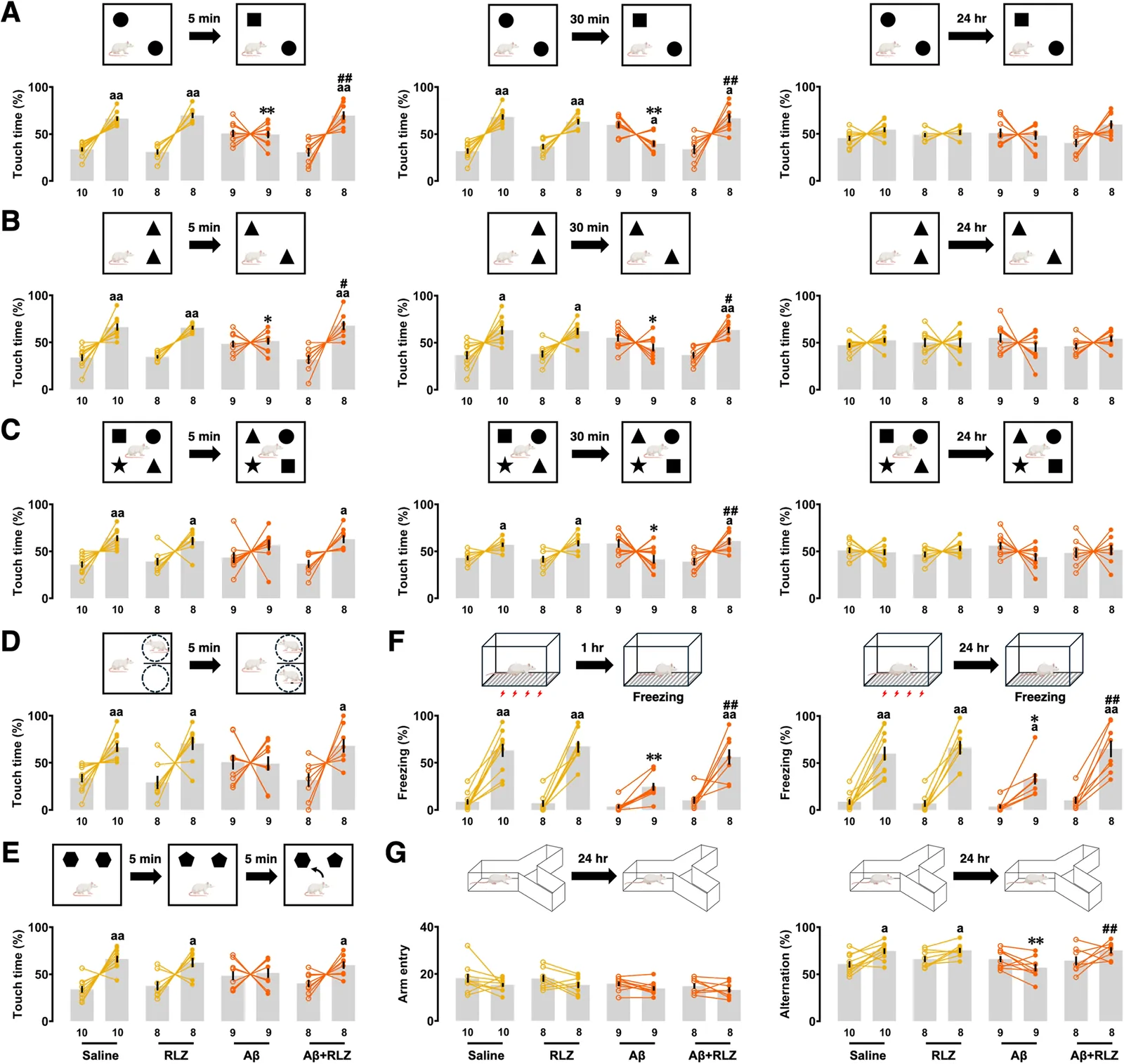
Riluzole improves Aβ_1-42_ oligomer–induced impairments across multiple hippocampus-dependent memory tasks. **A** Object recognition task showing the percentage of exploration time directed toward the novel versus familiar object at 5 min, 30 min, and 24 hr after training. **B** Object location task assessing preference for the novel spatial location at 5 and 30 min after training. **C** Object-in-place task evaluating associative spatial recognition memory at 5 and 30 min after training. **D** Social recognition task measuring interaction time with familiar and novel social targets. Open and closed circles indicate familiar and novel data, respectively. **E** Object recency task assessing temporal order memory by comparing exploration of recent (open) and old (closed) objects. **F** Contextual fear conditioning task showing freezing behavior during re-exposure to the conditioning chamber at 1 and 24 hr after training. **G** Y-maze spontaneous alternation task assessing spatial working memory; total arm entries and alternation performance are shown. Open and closed circles indicate training and retrieval data, respectively. Data were shown as mean ± SEM; sample sizes were indicated below each group. ^a^P < 0.05, ^aa^P < 0.01 vs familiar target or training; *P < 0.05, **P < 0.01 vs saline; ^#^P < 0.05, ^##^P < 0.01 vs Aβ

In the object recognition task (Fig. 2A), touch time of the novel object increased significantly in the saline (*t* = 7.288, *P* < 0.0001, paired t-test), RLZ (*t* = 6.858, *P* = 0.0002), and Aβ + RLZ groups (*t* = 4.263, *P* = 0.004). However, the Aβ group did not show an increase in touch time after a 5-min test interval (*t* = 0.155, *P* = 0.881). Aβ-induced impairment in object recognition memory improved by RLZ injection after 5 min. Thirty minutes later, the saline (t = 6.497, P = 0.0001), RLZ (t = 4.469, P = 0.0029), and Aβ + RLZ groups (t = 3.327, P = 0.013) continued to explore novel objects, while the Aβ group showed a reduced preference (t = 3.083, P = 0.015, paired t-test). Aβ-induced impairment improved with RLZ after 30 min. However, after 24 h, all groups spent a comparable amount of time exploring both objects.

In the object location task (Fig. 2B), after a 5-min interval, the saline (*t* = 4.518, *P* = 0.0015), RLZ (*t* = 8.792, *P* < 0.0001), and Aβ + RLZ groups (*t* = 3.800, *P* = 0.0067) – but not the Aβ_1-42_ group (*t* = 0.409, *P* = 0.6936, paired t-test) – preferentially explored the novel location. RLZ injection improved Aβ-induced impairment in object location memory after 5 min. A similar pattern was observed 30 min later, with significantly higher exploration time in the novel location in the saline (*t* = 3.100, *P* = 0.0127), RLZ (*t* = 3.186, *P* = 0.0154), and Aβ + RLZ groups (*t* = 3.3958, *P* = 0.0055), but not in the Aβ_1-42_ group (*t* = 1.277, *P* = 0.2376, paired t-test). Aβ-induced impairment was also improved in the Aβ + RLZ group after 30 min. However, after 24 h, no differences were detected among the groups.

In the object-in-place task (Fig. 2C), the saline (*t* = 4.484, *P* = 0.0015), RLZ (*t* = 2.586, *P* = 0.0361), and Aβ + RLZ groups (*t* = 2.746, *P* = 0.0165) showed increased touch time of the exchanged objects after 5 min, while the Aβ_1-42_ group did not (*t* = 1.152, *P* = 0.2826, paired t-test). After 30 min, touch time of the exchanged objects increased in the saline (*t* = 3.160, *P* = 0.0116), RLZ (*t* = 2.469, *P* = 0.0429), and Aβ + RLZ groups (*t* = 2.717, *P* = 0.0228), but not in the Aβ_1-42_ group (*t* = 1.862, *P* = 0.0996, paired t-test). Aβ-induced impairment in object association memory improved after 5- and 30-min intervals following RLZ injection. However, no significant differences were observed after 24 h.

In the social recognition task (Fig. 2D) the saline (*t* = 3.550, *P* = 0.0062), RLZ (*t* = 2.932, *P* = 0.0220), and Aβ + RLZ groups (*t* = 2.506, *P* = 0.0406) interacted more with the novel social target after a 5-min test interval, though the Aβ_1-42_ group did not (*t* = 0.095, *P* = 0.9263, paired t-test). In the object recency task (Fig. 2E), the time spent touching the old object was significantly longer in the saline (*t* = 4.649, *P* = 0.0012), RLZ (*t* = 2.524, *P* = 0.0396), and Aβ + RLZ groups (*t* = 2.451, *P* = 0.0440), after the second sampling phase, but not in the Aβ_1-42_ group (*t* = 0.312, *P* = 0.7632, paired t-test).

In the fear conditioning test (Fig. 2F), after 1 h post-training, a three-way ANOVA revealed main effects of training (*F*_1,62_ = 155.970, *P* < 0.0001), Aβ (*F*_1,62_ = 12.515, *P* = 0.0008) and riluzole (*F*_1,62_ = 7.882, *P* = 0.0067). Freezing time increased in the saline, RLZ, and Aβ + RLZ groups (all; *p* < 0.0001 vs. training) but not in the Aβ group (*p* = 0.1320 vs. training). Aβ impaired contextual memory (Aβ vs. saline; *p* < 0.0001), which improved after riluzole injection (Aβ vs. Aβ + RLZ; *p* = 0.0016). After 24 h, a three-way ANOVA revealed main effects of training (*F*_1,62_ = 146.670, *P* < 0.0001) and riluzole (*F*_1,62_ = 7.104, *P* = 0.0098) but no significant Aβ effect (*F*_1,62_ = 3.491, *P* = 0.0664). Freezing time increased in all groups (all; *P* < 0.0001 vs. training) including the Aβ group (*P* = 0.0111 vs. training), and the Aβ group showed partial impairment alleviated by RLZ injection (Aβ vs. Aβ + RLZ; *P* = 0.0068).

In the Y-maze task (Fig. 2G), arm entries tended to decrease after 24 h, though this change was not statistically significant (saline: *t* = 1.397, *P* = 0.1959; RLZ: *t* = 1.795, *P* = 0.1158; Aβ: *t* = 2.121, *p* = 0.0667; Aβ + RLZ: *t* = 1.140, *P* = 0.2920, paired t-test). Training increased the alternation ratio in the saline (*t* = 3.052, *P* = 0.0138) and RLZ groups (*t* = 2.829, *P* = 0.0254) but not in the Aβ (*t* = 2.289, *P* = 0.0514) and Aβ + RLZ groups after 24 h (*t* = 1.811, *P* = 0.1131, paired t-test). Two-way ANOVA revealed main effects of Aβ (*F*_1,31_ = 7.985, *P* = 0.0082) and riluzole (*F*_1,31_ = 8.969, *P* = 0.0054), as well as a significant interaction (*F*_1,31_ = 7.670, *P* = 0.0094). Aβ-induced impairment in spatial working memory (Aβ vs. saline; *P* = 0.0015) improved in the Aβ + RLZ group (Aβ vs. Aβ + RLZ; *P* = 0.0020). Together, these results demonstrate that riluzole broadly rescues Aβ_1-42_ oligomer–induced impairments across multiple hippocampus-dependent memory domains, without affecting exploratory activity or motivation.

### The effect of riluzole on Aβ_1−42_ oligomer deposition and clearance by astrocytes and microglia

**Fig. 3:**
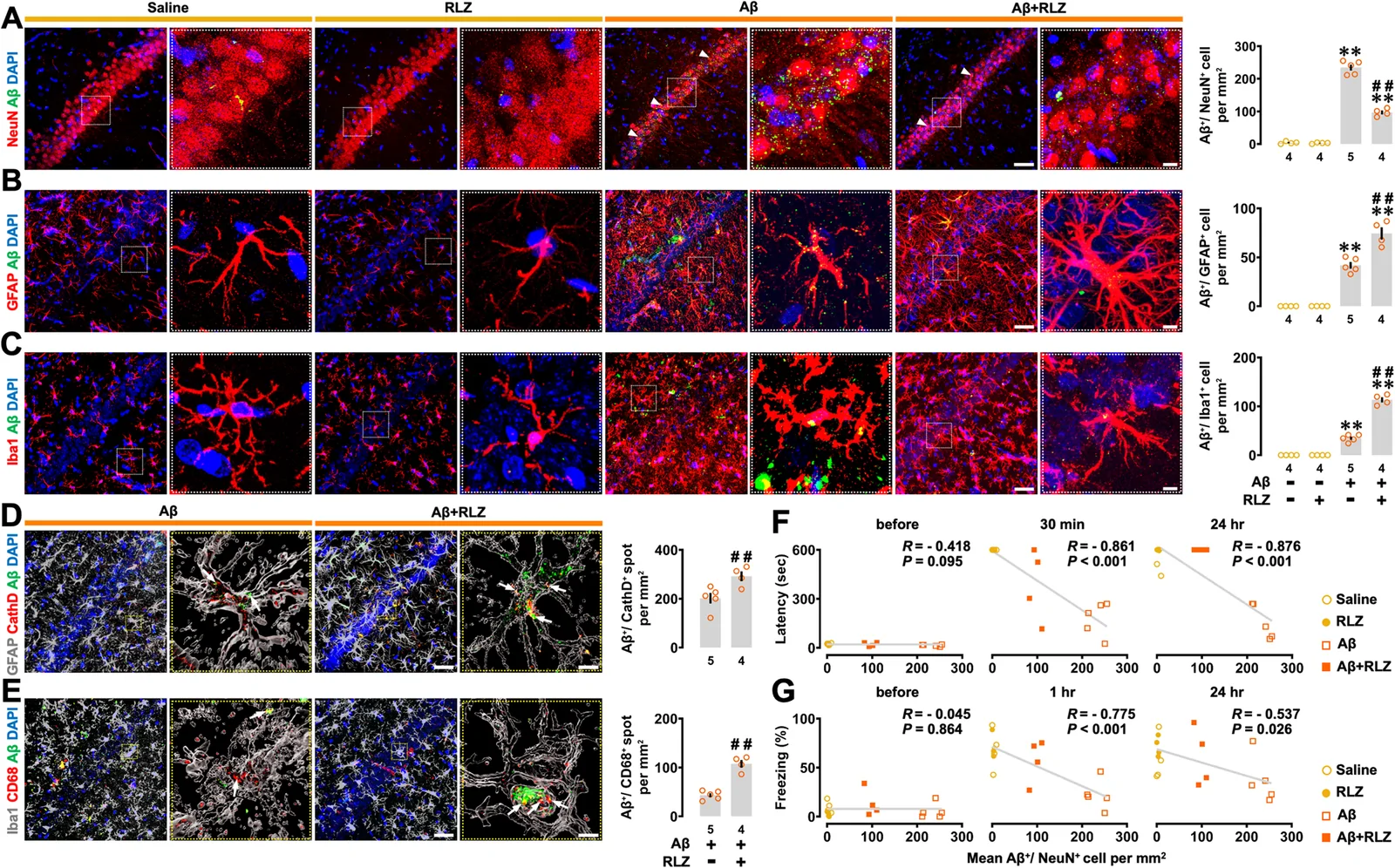
Riluzole alters the cellular distribution of Aβ_1-42_ oligomers in the dorsal CA1 region. **A** Representative super-resolution images and quantification of lecanemab-positive neurons per mm^2^ in the dorsal CA1 region. The number of Aβ⁺ neurons was reduced in the Aβ+RLZ group. **B**, **C** Representative super-resolution images and quantification of lecanemab-positive astrocytes and microglia. The number of Aβ-associated astrocytes (**B**) and microglia (**C**) were increased in the Aβ+RLZ group. **D**, **E** Representative super-resolution images and quantification of colocalized Aβ/Cathepsin D and Aβ/CD68 puncta per mm². The zoom-in images showed surface-rendered glial cells with masked lysosomal marker and Aβ. **F**, **G** Correlation analyses between learning performance and neuronal Aβ burden. Learning performance after training, but not before, was negatively correlated with mean number of Aβ^+^ neurons per animal in the IA task (**F**) and contextual fear conditioning (**G**). Scale bars: 40 μm with zoom-in 5 μm. Arrowheads indicate colocalized cells and arrows represent colocalized spots. Data were shown as mean ± SEM; sample sizes were indicated below each group. **P < 0.01 vs saline; ^##^P < 0.01 vs Aβ

First, we examined the effects of riluzole on Aβ deposition in neurons and processing by astrocytes and microglia. Aβ/NeuN^+^ cells increased markedly in the Aβ group and decreased in the Aβ + RLZ group (Fig. 3A, Aβ: *F*_*1,13*_ = 713.817, *P* < 0.0001; riluzole: *F*_*1,13*_ = 123.194, *P* < 0.0001; interaction: *F*_*1,13*_ = 123.194, *P* < 0.0001). Next, the number of Aβ/GFAP^+^ cells were increased in the Aβ group and increased further in the Aβ + RLZ group (Fig. 3B, Aβ: *F*_*1,13*_ = 267.852, *P* < 0.0001; riluzole: *F*_*1,13*_ =21.014, *P* = 0.0005; interaction: *F*_*1,13*_ =21.014, *P* = 0.0005). Similarly, the number of Aβ/Iba1^+^ cells were increased in the Aβ group and was further elevated in the Aβ + RLZ group (Fig. 3C, Aβ: *F*_*1,13*_ = 555.839, *P* < 0.0001; riluzole: *F*_*1,13*_ = 156.550, *P* < 0.0001; interaction: *F*_*1,13*_ = 156.550, *P* < 0.0001). Surface-rendered 3D reconstructions further demonstrated that riluzole increased internalized Aβ within astrocytes (Video S1 and S2) and microglia with a relative decrease in unengulfed Aβ (Figure S2). Therefore, riluzole reduced the neuronal Aβ while enhancing Aβ binding and engulfing by astrocytes and microglia.

Next, we examined the effect of riluzole on Aβ degradation within astrocytes and microglia using lysosomal markers, Cathepsin D and CD68 respectively. Colocalized Aβ/CathD (Fig. 3D, *t* = 2.982, *P* = 0.0207, unpaired t-test) and Aβ/CD68 spots were increased in the Aβ + RLZ group (Fig. 3E, *t* = 7.766, *P* = 0.0001, unpaired t-test). These data highlight that riluzole enhances Aβ internalization and lysosome-mediated degradation by astrocytes and microglia.

To further establish a brain–behaviour link, we examined correlations between contextual learning performance and neuronal Aβ burden of individual animals. No significant correlation was detected prior to the IA task (*R* = − 0.418, *P* = 0.0951). In contrast, a strong negative correlation emerged between the number of Aβ/NeuN^+^ cells and IA performance measured at both 30 min (*R* = − 0.861, *P* < 0.0001) and 24 h (*R* = − 0.876, *P* < 0.0001) after training (Fig. 3F).

A similar relationship was observed in the contextual fear conditioning paradigm (Fig. 3G). Before shock exposure, freezing behavior did not correlate with neuronal Aβ burden (*R* = − 0.045, *P* = 0.8639). However, significant negative correlations were detected between the number of Aβ/NeuN^+^ cells and freezing behaviour at 1 h (*R* = − 0.775, *P* = 0.0003) and 24 h (*R* = − 0.537, *P* = 0.0262) after conditioning. Together, these results indicate that reductions in neuronal Aβ burden—consistent with enhanced glial-mediated Aβ clearance—are tightly associated with improved hippocampus-dependent learning and memory performance.

### Effect of riluzole on Aβ_1-42_ oligomer-induced neuronal apoptosis, spine loss and alterations in spine morphology

**Fig. 4:**
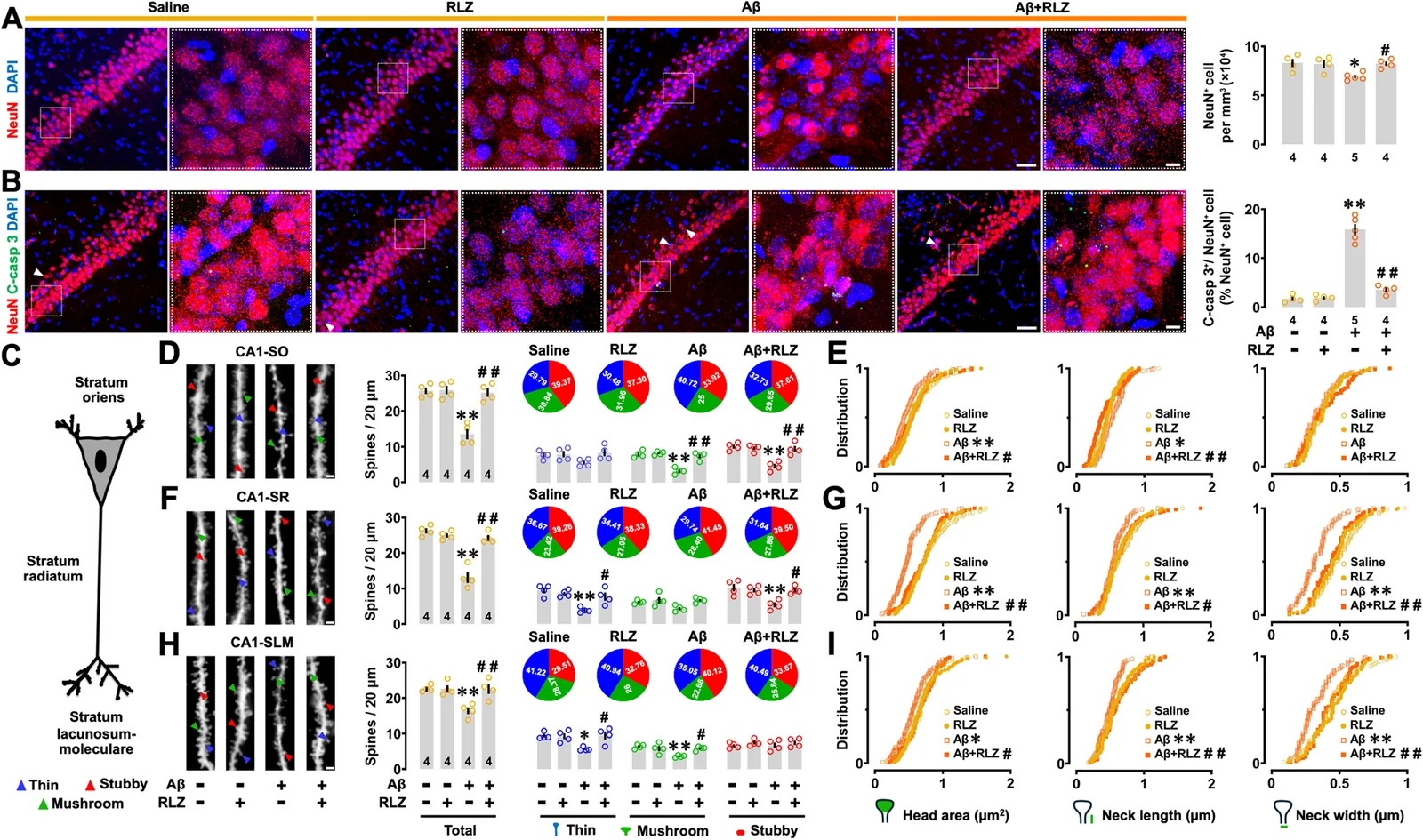
Effects of riluzole on neuronal survival, spine number and morphology in the dorsal CA1 region. **A**, **B** Representative super-resolution images and quantification of NeuN⁺ cells (**A**) and cleaved caspase-3/NeuN⁺ cells (**B**) in the dorsal CA1. **C** Illustration of a CA1 pyramidal neuron with its sublayer. **D**, **F**, **H** Representative Golgi-stained images and quantification of dendritic spine density (spines per 20 μm) in the stratum oriens (**D**), stratum radiatum (**F**), and stratum lacunosum-moleculare (H). Pie charts indicate the proportion of thin, mushroom, and stubby spines in each group. **E**, **G**, **I** Cumulative distributions of spine head area, neck length, and neck width for individual spines in the stratum oriens (**E**), stratum radiatum (**G**), and stratum lacunosum-moleculare (**I**). Arrowheads indicate colocalized cells. Golgi-stained dendrites were pseudocolored in gray. Colored arrowheads indicate spine types. Scale bars: 40 μm with zoom-in 5 μm (**A**, **B**) and 2 μm (**D**, **F**, **H**). Data were shown as mean ± SEM; sample sizes were indicated below each group. *P < 0.05, **P < 0.01 vs saline; ^#^P < 0.05, ^##^P < 0.01 vs Aβ

We examined the effects of riluzole on Aβ_1-42_ oligomer-induced neuronal apoptosis and dendritic degeneration. First, Aβ_1-42_ oligomers reduced the number of NeuN^+^ cells; but this effect was reversed in the Aβ + RLZ group (Fig. 4A, Aβ: *F*_*1,13*_ =5.199, *P* = 0.0414; riluzole: *F*_*1,13*_ =4.161, *P* = 0.0622; interaction: *F*_*1,13*_ = 5.566, *P* = 0.0346). Similarly, Aβ-induced reduction in MAP2 intensity was reversed in the Aβ + RLZ group (Figure S5, Aβ: *F*_*1,13*_ = 180.388, *P* < 0.0001; riluzole: *F*_*1,13*_ = 101.480, *P* < 0.0001; interaction: *F*_*1,13*_ = 99.006, *P* < 0.0001). Conversely, the increased number of cleaved caspase-3/NeuN^+^ cells found in the Aβ group was reduced in the Aβ + RLZ group (Fig. 4B, Aβ: *F*_*1,13*_ = 112.271, *P* < 0.0001; riluzole: *F*_*1,13*_ = 65.991, *P* < 0.0001; interaction: *F*_*1,13*_ = 70.430, *P* < 0.0001). These data suggest that riluzole mitigates Aβ_1-42_ oligomer-induced neuronal apoptosis and dendritic degeneration.

Next, we performed Golgi staining and analyzed spine number as well as morphology. Figure S6 showed an overview image of pyramidal neurons with zoom-in representative dendritic spines across the CA1 sublayers. In the stratum oriens (Fig. 4D), the loss of total spines, as well as mushroom and stubby spines, induced by Aβ was reversed in the Aβ + RLZ group. This was accompanied by the reversal of smaller Aβ-induced spine heads and shorter necks of individual spines in the Aβ + RLZ group (Fig. 4E). In the stratum radiatum (Fig. 4F), Aβ reduced the total spine density without affecting the mushroom spine type. These effects were reversed in the Aβ + RLZ group. Furthermore, riluzole restored the smaller heads, shorter necks and narrower necks induced by Aβ (Fig. 4G). In the stratum lacunosum-moleculare (Fig. 4H), Aβ induced loss of total spines, specifically thin and mushroom spines. These were recovered in the Aβ + RLZ group. In the Aβ + RLZ group, riluzole reversed the smaller heads, shorter necks, and narrower necks induced by Aβ (Fig. 4I). Overall, our findings demonstrate that riluzole reverses the effects of Aβ_1-42_ oligomers on spine loss and alterations in spine morphology (see Table S2 for statistical results).

### Effect of riluzole on Aβ_1-42_ oligomer-induced complement-mediated synaptic elimination by microglia

**Fig. 5:**
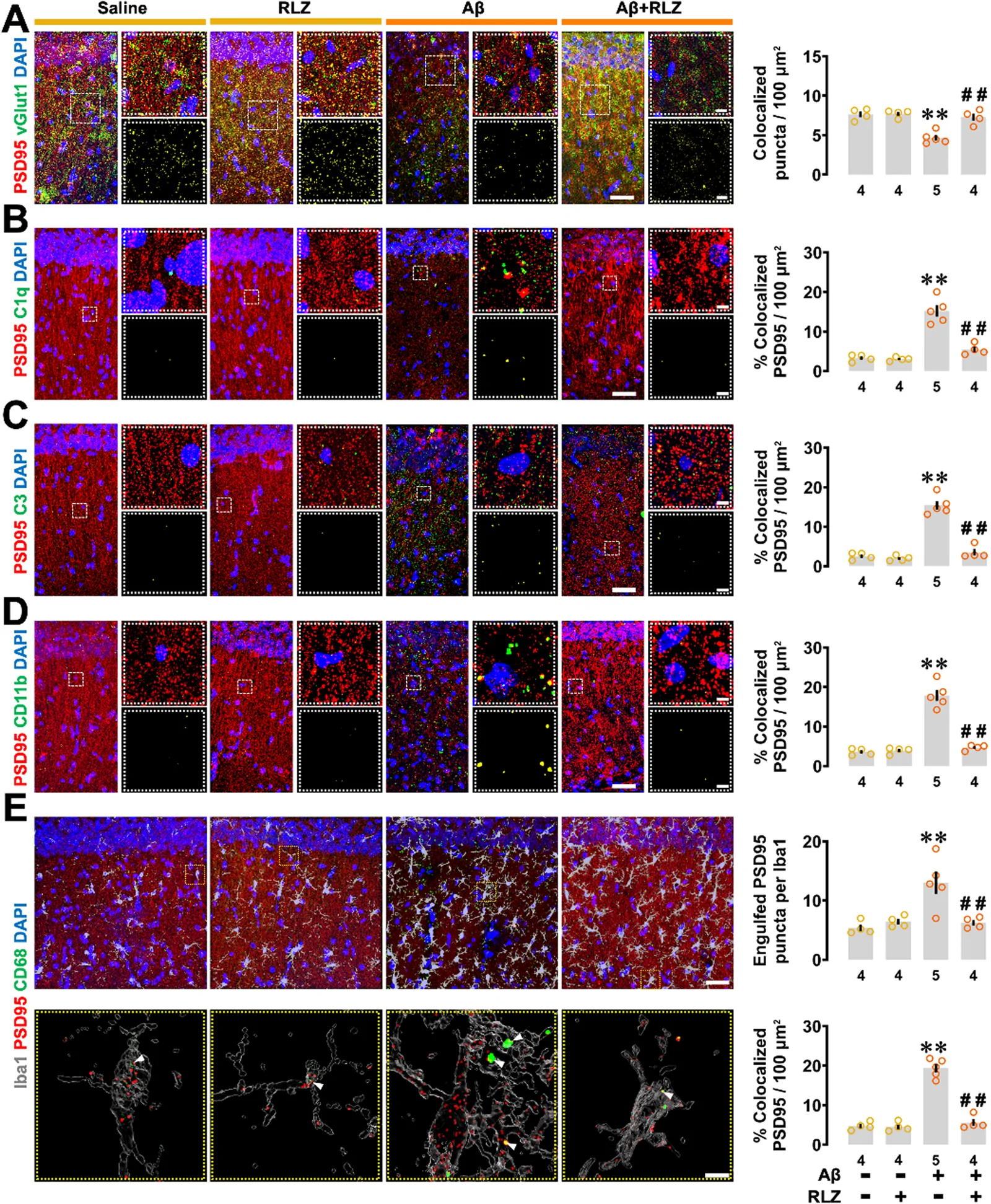
Effects of riluzole on synaptic markers and complement-mediated synaptic pruning by microglia in the dorsal CA1 region. **A** Representative super-resolution images and colocalized vGlut1/PSD95 puncta per 100 μm² in the stratum radiatum of the dorsal CA1. **B**, **C**, **D** Representative images and percentage of colocalized C1q/PSD95 (**B**), C3/PSD95 (**C**) and CD11b/PSD95 (**D**) puncta per 100 μm². **E** Representative super-resolution images (top) with zoom-in images (bottom) showing surface-rendered microglia with masked lysosomal marker and PSD95. Number of PSD95 puncta engulfed by microglia and percentage of colocalized CD68/PSD95 puncta per 100 μm² were quantified. Yellow areas indicate colocalized puncta and arrowheads indicate engulfed PSD95 puncta colocalized with CD68. Scale bars: 40 μm with zoom-in 5 μm. Data were shown as mean ± SEM; sample sizes were indicated below each group. **P < 0.01 vs saline; ^##^P < 0.01 vs Aβ

To determine whether riluzole modulates synaptic integrity under Aβ oligomer exposure, we first examined synaptic puncta in the dorsal CA1 region. To investigate how riluzole rescued Aβ-induced synaptic loss, we quantified the number of colocalized vGlut1/PSD95 puncta. In the stratum radiatum, the number of synaptic puncta decreased in the Aβ group, which was recovered in the Aβ + RLZ group (Fig. 5A, Aβ: *F*_*1,13*_ = 22.410, *P* = 0.0004; riluzole: *F*_*1,13*_ = 13.957, *P* = 0.0025; interaction: *F*_*1,13*_ = 12.204, *P* = 0.0040). Similar changes were also observed in the stratum lacunosum-moleculare (Figure S7A). However, the reduction in synaptic puncta was attributable to the loss of the postsynaptic protein PSD95 rather than the presynaptic marker vGlut1 (Figure S7B; Table S3 for statistical results).

Next, we examined complement-mediated synaptic engulfment by microglia. Firstly, C1q/PSD95 colocalization increased in the Aβ group but remained at the control level in the Aβ + RLZ group (Fig. 5B, Aβ: *F*_*1,13*_ = 56.491, *P* < 0.0001; riluzole: *F*_*1,13*_ = 27.337, *P* = 0.0002; interaction: *F*_*1,13*_ = 24.623, *P* = 0.0003). Subsequently, increased C3/PSD95 colocalization found in the Aβ group was reversed in the Aβ + RLZ group (Fig. 5C, Aβ: *F*_*1,13*_ = 80.479, *P* < 0.0001; riluzole: *F*_*1,13*_ = 60.032, *P* < 0.0001; interaction: *F*_*1,13*_ = 49.891, *P* < 0.0001). Similarly, CD11b/PSD95 colocalization was also increased in the Aβ group which was reduced in the Aβ + RLZ group (Fig. 5D, Aβ: *F*_*1,13*_ = 67.803, *P* < 0.0001; riluzole: *F*_*1,13*_ = 49.693, *P* < 0.0001; interaction: *F*_*1,13*_ = 55.038, *p* < 0.0001). Consistently, the number of PSD95 puncta within microglia was elevated in the Aβ group and restored in the Aβ + RLZ group (Fig. 5E, upper panel; Aβ: *F*_*1,13*_ = 9.429, *P* = 0.0089; riluzole: *F*_*1,13*_ = 5.590, *P* = 0.0343; interaction: *F*_*1,13*_ = 10.449, *P* = 0.0065). CD68/PSD95 colocalization showed a similar pattern (Fig. 5E, lower panel; Aβ: *F*_*1,13*_ = 88.474, *P* < 0.0001; riluzole: *F*_*1,13*_ = 69.275, *P* < 0.0001; interaction: *F*_*1,13*_ = 65.407, *P* < 0.0001). These results indicate that riluzole attenuates Aβ_1-42_ oligomer–induced synaptic loss in parallel with reduced complement-associated microglial synaptic elimination.

### Effect of riluzole on Aβ_1−42_ oligomer–induced astrocyte recruitment, morphology, and marker expression

**Fig. 6:**
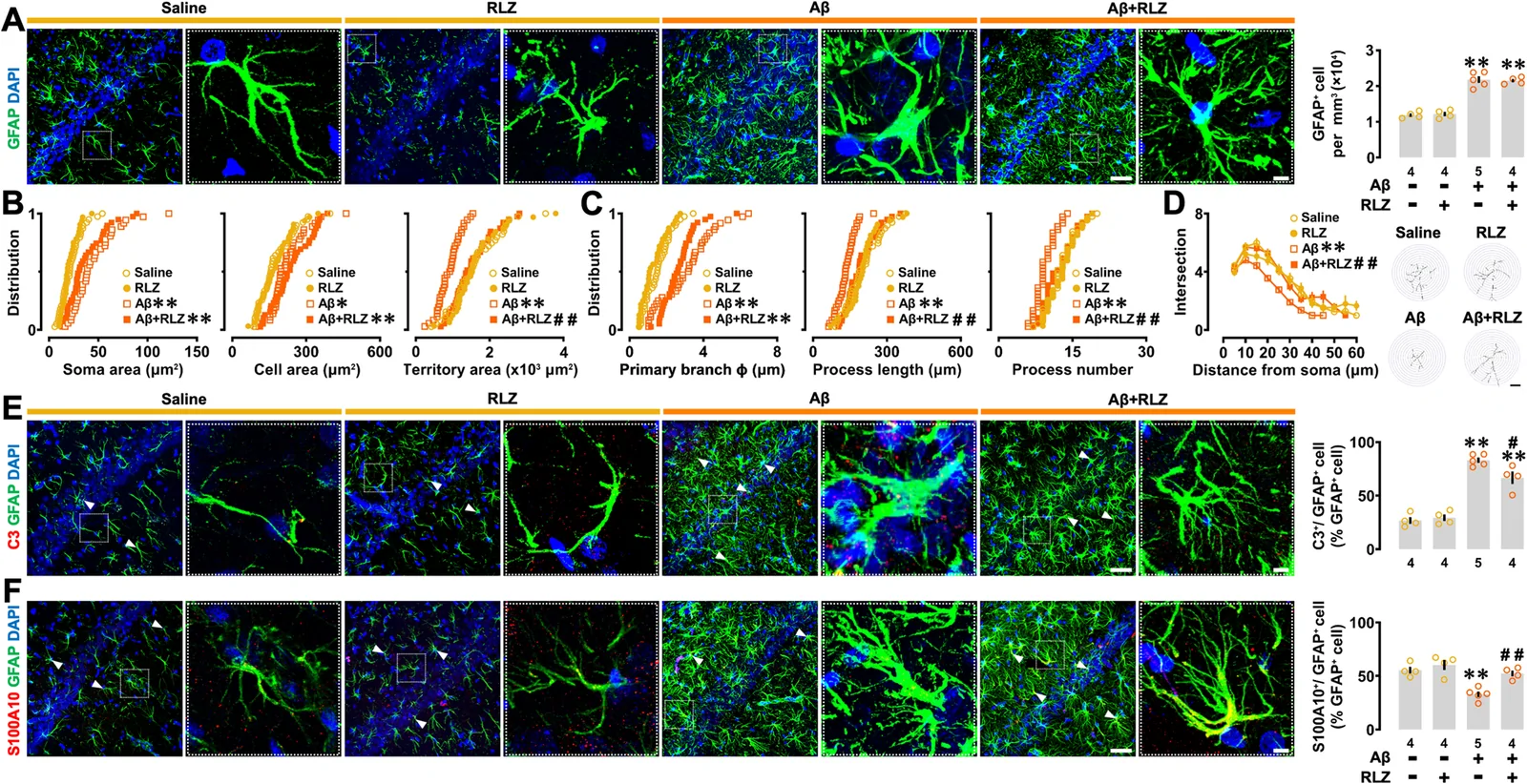
Effects of riluzole on astrocyte number, morphology, and polarization in the dorsal CA1 region. **A** Representative super-resolution images and quantification of GFAP⁺ cells per mm^3^ in the dorsal CA1 region. **B** Quantification of size- and shape-related astrocytic parameters. **C** Quantification of branching-related parameters. **D** Sholl analysis (left) and representative reconstructed astrocytic skeletons (right). Intersections were quantified at 5-µm intervals from the soma. **E**, **F** Representative super-resolution images and percentage of C3/GFAP⁺ (**E**) and S100A10/GFAP⁺ (**F**) cells per mm². Arrowheads indicate double-positive cells. Scale bars: 40 μm with zoom-in 5 μm (**A**, **E**, **F**) and 20 μm (**D**). Data were shown as mean ± SEM; sample sizes were indicated below each group. *P < 0.05, **P < 0.01 vs saline; ^#^P < 0.05, ^##^P < 0.01 vs Aβ

Given the close involvement of astrocytes in complement signalling and synaptic regulation, we next assessed astrocytic responses in the dorsal CA1 region. Two-way ANOVA revealed that Aβ_1-42_ oligomers triggered robust recruitment of GFAP⁺ astrocytes, which persisted in the Aβ + RLZ group (Fig. 6A, Aβ: *F*_*1,13*_ = 188.683, *P* < 0.0001; riluzole: *F*_*1,13*_ = 0.0007, *P* = 0.9785; interaction: *F*_*1,13*_ = 0.184, *p* = 0.6746).

Regarding morphology, soma and cell area increased in both the Aβ and Aβ + RLZ groups (Fig. 6B). Territory area decreased in the Aβ group but reversed in the Aβ + RLZ group. Analysis of branching complexity showed increased primary branch thickness in both groups (Fig. 6C). In contrast, Aβ-induced reduction in total process length and process number was reversed in the Aβ + RLZ group. Sholl analysis confirmed recovery of branching complexity (Fig. 6D).

Finally, percentage of C3⁺/GFAP⁺ astrocytes were increased in the Aβ group and significantly reduced in the Aβ + RLZ group, whereas S100A10⁺/GFAP⁺ astrocytes showed the opposite pattern (Fig. 6E, Aβ: *F*_*1,13*_ = 346.541, *P* < 0.0001; riluzole: *F*_*1,13*_ = 3.391, *P* = 0.0885; interaction: *F*_*1,13*_ = 7.488, *P* = 0.0170. Figure 6F, Aβ: *F*_*1,13*_ = 34.244, *P* < 0.0001; riluzole: *F*_*1,13*_ = 17.080, *P* = 0.0012; interaction: *F*_*1,13*_ = 6.013, *P* = 0.0291). Riluzole shifted astrocytes toward neuroprotective phenotype, markedly reducing A1 astrocytes and increasing A2 astrocytes (Figure S8).

Together, these findings show that riluzole preserves astrocytic recruitment while normalizing astrocytic morphology and modulating marker expression.

## Effect of riluzole on Aβ_1−42_ oligomer–induced microglial recruitment, morphology, and functional marker expression

**Fig. 7:**
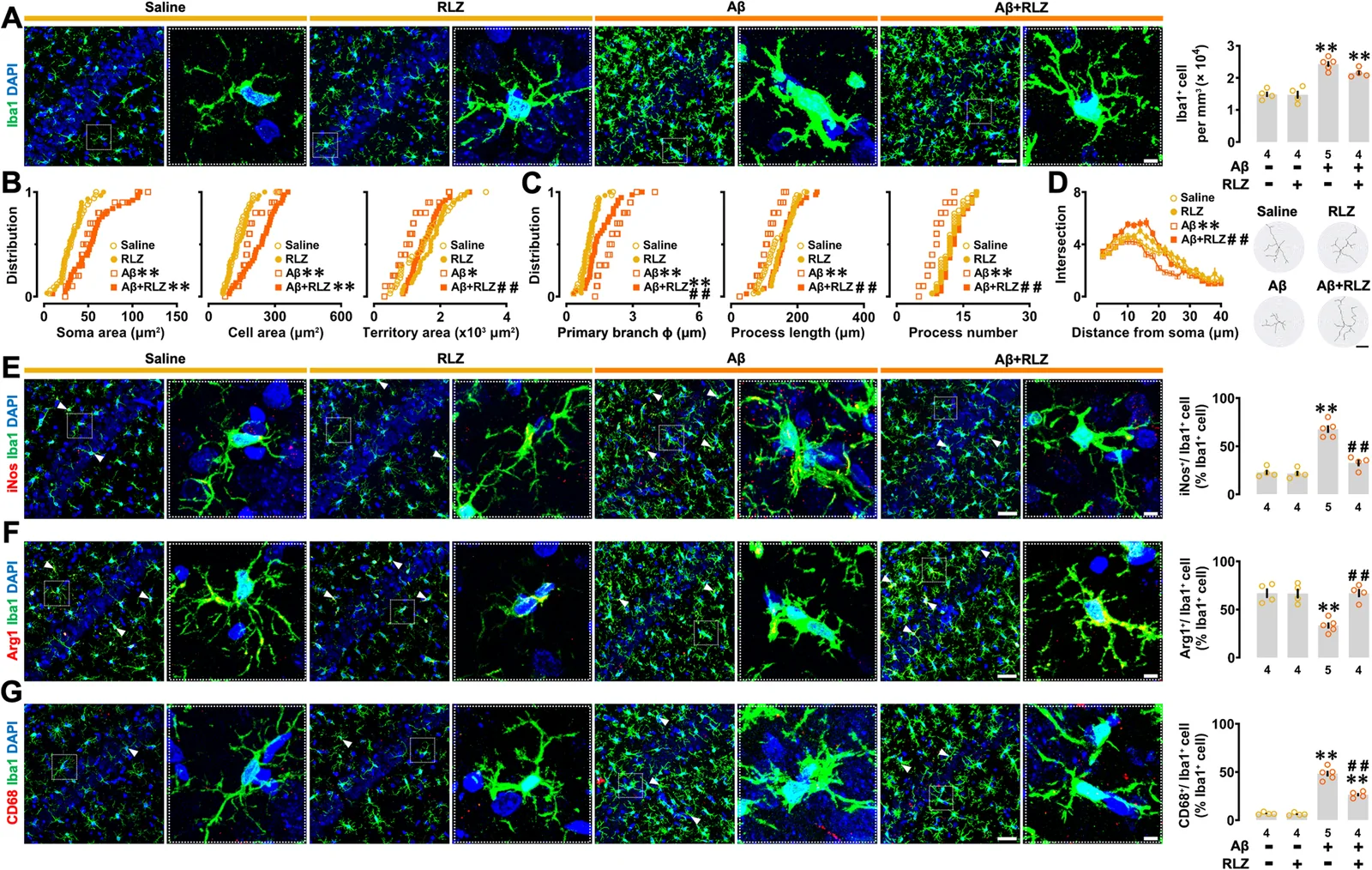
Effects of riluzole on microglial number, morphology, and marker expression in the dorsal CA1 region. **A** Representative super-resolution images and quantification of Iba1⁺ cells per mm^3^ in the dorsal CA1 region. **B** Quantification of size- and shape-related microglial parameters. **C** Quantification of branching-related parameters. **D** Sholl analysis (left) and representative reconstructed microglial skeletons (right). Intersections were quantified at 2-µm intervals from the soma. **E**, **F**, **G** Representative super-resolution images and percentage of iNos/Iba1⁺ (**E**), Arg1/Iba1⁺ (**F**), and CD68/Iba1⁺ (**G**) cells per mm². Arrowheads indicate double-positive cells. Scale bars: 40 μm with zoom-in 5 μm (**A**, **E**–**G**) and 20 μm (**D**). Data were shown as mean ± SEM; sample sizes were indicated below each group. *P < 0.05, **P < 0.01 vs saline; ^##^P < 0.01 vs Aβ

We then examined whether microglial responses were similarly modulated by riluzole. Aβ_1-42_ oligomers significantly increased Iba1⁺ microglial density, which persisted in the Aβ + RLZ group (Fig. 7A, Aβ: *F*_*1,13*_ = 76.342, *P* < 0.0001; riluzole: *F*_*1,13*_ = 2.399, *P* = 0.1454; interaction: *F*_*1,13*_ = 1.932, *P* = 0.1878).

Morphological analysis revealed increased soma and cell area in both groups (Fig. 7B). Aβ-induced reductions in territory size and increases in cell-to-territory area ratio were partially normalized in the Aβ + RLZ group. Branching analysis showed partial recovery of increased primary branch thickness in the Aβ + RLZ group. In contrast, reductions in process length and process number in the Aβ group were reversed by riluzole (Fig. 7C). Sholl analysis confirmed recovery of branching complexity (Fig. 7D).

Consistent with these structural changes, riluzole reduced the proportion of iNos⁺/Iba1⁺ microglia (Fig. 7E, Aβ: *F*_*1,13*_ = 424.045, *P* < 0.0001; riluzole: *F*_*1,13*_ = 198.819, *P* < 0.0001; interaction: *F*_*1,13*_ = 183.408, *P* < 0.0001) while increasing Arg1⁺/Iba1⁺ cells and decreasing CD68⁺/Iba1⁺ microglia (Fig. 7F, Aβ: *F*_*1,13*_ = 72.003, *P* < 0.0001; riluzole: *F*_*1,13*_ = 79.500, *P* < 0.0001; interaction: *F*_*1,13*_ = 96.376, *P* < 0.0001. Figure 7G, Aβ: *F*_*1,13*_ = 237.362, *P* < 0.0001; riluzole: *F*_*1,13*_ = 31.730, *P* < 0.0001; interaction: *F*_*1,13*_ = 28.042, *P* = 0.0001). Riluzole shifted microglia toward neuroprotective phenotype, markedly reducing M1 microglia and increasing M2 microglia (Figure S9).

Additionally, Figure S10 shows the percentage of different morphological types of microglia in each group (see Table S4 for statistical results). These findings suggest that riluzole sustains microglial recruitment while modulating morphology and marker expression.

## Discussion

### Riluzole improves Aβ_1−42_ oligomer–induced memory impairment by enhancing glial-mediated Aβ clearance

In the present study, we show that riluzole ameliorates Aβ_1−42_ oligomer–induced cognitive impairment and attenuates Aβ pathology by promoting glial-mediated Aβ clearance. Importantly, across individual animals, learning performance was strongly and negatively correlated with neuronal Aβ burden, providing a direct behavioral link to glial-mediated Aβ handling. Rather than suppressing glial activation, riluzole reshaped astrocytic and microglial responses toward functional states associated with enhanced Aβ uptake, degradation, and synaptic preservation.

Aβ_1−42_ oligomers disrupt glutamatergic signaling [6] and induce neuronal hyperexcitability [5, 7], ultimately leading to cognitive impairment [8, 9]. Importantly, Aβ-induced neurotoxicity is tightly linked to glutamatergic dysfunction [10], which in turn promotes amyloid precursor protein (APP) processing and further Aβ release [37]. This reciprocal relationship suggests that interventions stabilizing glutamatergic signaling may indirectly facilitate Aβ clearance. In this context, the cognitive benefits of riluzole observed here are likely attributable, at least in part, to enhanced removal of Aβ rather than reduced production.

Consistent with our findings, previous studies have shown that riluzole improves cognitive performance in both injection [9] and transgenic AD models with reduced amyloid burden [19, 38, 39]. Importantly, the cognitive benefits of riluzole were most pronounced during the 5 and 30-min intervals and diminished at the 24-h time point in the object-based memory tasks. These findings suggest a transient effect of riluzole, potentially underscoring sustained administration to maintain long-term efficacy. However, contextual memory remained stable at 24 h. This differential outcome may reflect the influence of task-related factors whereby distinct forms of memory retention exhibit varying susceptibility to decay over time.

Because riluzole does not alter amyloidogenic APP processing [39], these effects are most parsimoniously explained by enhanced Aβ clearance mechanisms. Despite their robust response, the precise contribution of each glial population to Aβ removal has not been fully resolved. Microglia exhibit greater uptake of fibrillar Aβ [40] whereas astrocytes preferentially take up oligomeric Aβ [41]. However, synergistic cooperation promotes more efficient Aβ clearance [42, 43]. Consistent with this cooperative model [43], riluzole enhances complementary glial phagocytosis rather than selectively activating a single cell population.

Microglial uptake of Aβ is mediated by multiple pattern-recognition and scavenger receptors [44, 45]. Among these, complement receptor 3 (CR3), composed of CD11b and CD18 subunits, is a potent phagocytic receptor that plays a key role in microglial Aβ clearance [46–48]. The increased colocalization of CD11b with Aβ observed in our study (Figure S6) therefore suggests that riluzole enhances CD11b-dependent recognition and uptake of Aβ by microglia.

Efficient Aβ clearance by glial cells requires not only uptake but also lysosomal degradation [43, 48, 49]. Supporting this, we found greater Aβ colocalization with lysosomes inside glial cells. Recent work further indicates that microglial metabolic state critically influences phagocytic efficiency, with enhanced mitochondrial oxidative phosphorylation supporting effective Aβ degradation [50]. Given that riluzole increases cerebral glucose oxidative metabolism [51], it is plausible that riluzole facilitates microglial Aβ clearance by promoting lysosome-dependent degradative capacity.

### Riluzole preserves neuronal survival and dendritic architecture in Aβ pathology

In the present study, riluzole attenuated Aβ oligomer–induced neuronal apoptosis, dendritic degeneration, and dendritic spine loss across hippocampal subregions. Aβ-induced excitotoxicity is known to cause excessive Ca²⁺ influx and mitochondrial dysfunction, ultimately leading to neuronal death [12]. Riluzole exerts neuroprotective effects by reducing presynaptic glutamate release [19] and enhancing glutamate uptake [18], thereby limiting excitotoxic stress. Consistent with previous reports in diverse disease models [20, 52, 53], our findings indicate that riluzole effectively protects neurons from Aβ-induced apoptotic degeneration.

Dendritic degeneration and spine loss are closely associated with cognitive dysfunction in AD [54]. Because dendritic architecture determines the integrative and electrical properties of neurons, dendritic simplification contributes to network hyperexcitability [55] and impaired information processing [14]. Moreover, dendritic dystrophy and spine loss disrupt synaptic signaling and plasticity by reducing dendritic complexity and synaptic connectivity [56]. The preservation of dendritic structure observed following riluzole treatment therefore provides a plausible structural basis for the recovery of hippocampus-dependent memory.

Both natural and synthetic Aβ species induce robust dendritic spine loss [57, 58]. Although selective elimination of specific spine subtypes, such as mushroom spines, has been reported [59], our data indicate a largely non-selective reduction in spine density. Notably, riluzole has been shown to promote clustering of thin spines and enhance spine-to-spine interactions, thereby increasing circuit efficiency through synaptic input summation [22]. These effects provide a potential mechanism by which riluzole counteracts Aβ-induced spine loss and stabilizes synaptic transmission.

Activity-dependent synaptic plasticity is accompanied by coordinated spine remodeling, including spine head enlargement and neck shortening, which facilitate glutamatergic receptor accumulation and intracellular signaling [60, 61]. In contrast, Aβ exposure induces immature spine phenotypes and aberrant morphological changes [62, 63]. The restoration of dendritic spine density and morphology observed in our study, together with previous evidence that riluzole normalizes synapse-related gene expression [23] and rescues Aβ-induced LTP impairment [9], supports a model in which riluzole stabilizes synaptic structure and function under Aβ challenge.

### Riluzole limits complement-dependent synaptic elimination by microglia

Cognitive decline in AD correlates more strongly with soluble Aβ oligomer–induced synaptic loss than with plaque burden [6]. Aβ oligomers promote synapse elimination through activation of the classical complement cascade, a process that preferentially targets postsynaptic elements and coincides with dendritic spine loss [64, 65]. This aberrant pruning mechanism is increasingly recognized as a key contributor to memory impairment in AD.

Aβ oligomers activate complement system in a C1q-dependent manner, leading to microglial engulfment of synapses via C3–CR3 signaling [65, 66]. Mechanistically, C1q accumulation at vulnerable synapses promotes C3 deposition and subsequent activation of microglial C3 receptors, leading to excessive synaptic engulfment by microglia [67]. Notably, complement signaling also participates in Aβ clearance, and recent in vivo studies demonstrate that selective inhibition of C1q preserves synapses while allowing efficient Aβ removal [68]. These findings highlight a functional dissociation within the complement system which differentially regulates amyloid and synaptic phagocytosis.

Both excitotoxic stress and Aβ accumulation upregulate C3 expression and amplify neuroinflammatory signaling [65, 69]. By restoring glutamate homeostasis and reducing neuronal Aβ burden, riluzole may dampen excessive complement activation while preserving microglial phagocytic capacity. Consistent with this notion, we observed that riluzole reduced C1q- and C3-associated synaptic puncta and limited synaptic phagocytosis, without suppressing microglial recruitment. However, whether these recovered synapses exhibit synaptic plasticity requires electrophysiological validation.

Together, our data support a model in which riluzole uncouples complement-mediated Aβ clearance from synaptic elimination. By constraining aberrant C1q/C3–dependent pruning while maintaining microglial engagement with amyloid, riluzole preserves synaptic integrity and contributes to the recovery of hippocampal memory performance.

### Riluzole preserves astrocyte recruitment while promoting functional and phenotypic remodeling toward a neuroprotective state.

In contrast to studies reporting suppression of astrocytic activation, we found that riluzole maintained astrocyte recruitment in response to Aβ oligomers. Astrocyte accumulation around amyloid deposits is increasingly recognized as a protective response, as genetic deletion of astrocytic intermediate filaments exacerbates amyloid deposition and reduces astrocyte branching and plaque coverage [70]. These findings underscore the importance of astrocyte recruitment and physical engagement with amyloid pathology in constraining plaque expansion.

However, astrocyte recruitment alone is insufficient to confer neuroprotection. Reactive A1 astrocytes, characterized by elevated C3 expression, lose key homeostatic functions, including aquaporin-4 (AQP4) polarization, which is essential for glymphatic Aβ clearance [71, 72]. Moreover, chronic astrocytic C3 release impairs microglial Aβ phagocytosis and amplifies neuroinflammatory signaling [73]. Consistently, genetic or pharmacological suppression of C3 reduces inflammatory mediators while facilitating microglial-mediated Aβ clearance [74, 75].

In contrast, neuroprotective A2 astrocytes secrete trophic factors and anti-inflammatory cytokines that support neuronal survival and promote amyloid clearance [76, 77]. For example, astrocyte-derived interleukin-33 enhances microglial Aβ phagocytosis, while A2 polarization is associated with upregulation of Aβ-degrading enzymes such as neprilysin and scavenger receptors including CD36 [75, 78]. Our observation also indicates a phenotypic shift toward a neuroprotective astrocyte profile, rather than a suppression of astrocytic activation per se.

Astrocyte morphology further reflects and reinforces functional state. Simplified astrocytic arbors are associated with elevated C3 expression and increased amyloid burden [71, 79], whereas highly branched astrocytes establish extensive plaque contacts and are linked to reduced amyloid accumulation [70]. The restoration of astrocytic branching complexity and territory size observed with riluzole treatment therefore likely provides a structural substrate that supports enhanced Aβ handling and local synaptic protection.

Collectively, these findings suggest that riluzole sustains astrocyte recruitment while promoting functional and morphological remodeling of reactive astrocytes toward a neuroprotective state. This coordinated remodeling may enhance astrocyte–microglia cooperation in amyloid clearance and contribute to the preservation of hippocampal circuit integrity. 

### Riluzole preserves microglial recruitment while promoting a neuroprotective, phagocytosis-competent microglial state.

In contrast to reports describing suppression of microglial accumulation, we found that riluzole sustained microglial recruitment in response to Aβ oligomers. Although the role of microglia in amyloid pathology remains controversial, accumulating evidence supports a protective function under specific activation states. For instance, experimental depletion of microglia increases plaque size, whereas activation of TREM2 (Triggering receptor expressed on myeloid cells 2) enhances microglial clustering around plaques and reduces amyloid burden [80, 81]. These findings suggest that appropriate microglial recruitment can constrain amyloid plaque expansion.

Importantly, the functional outcome of microglial activation depends on their inflammatory state. Proinflammatory cytokine production is inversely correlated with the capacity of microglia to clear Aβ and can indirectly enhance amyloid deposition by upregulating β-secretase expression [44, 82, 83]. In contrast, microglia biased toward an anti-inflammatory, M2-like phenotype exhibit enhanced Aβ uptake and phagocytic efficiency [78, 82, 84]. Thus, microglial activation per se is not detrimental; rather, the balance between inflammatory and phagocytic states determines functional outcome.

CD68 reactivity has been shown to correlate positively with C3-expressing A1 astrocytes, amyloid burden, and cognitive impairment in AD [71, 74, 85, 86]. This association suggests that inefficient or dysregulated phagocytic activation may contribute to aberrant synaptic elimination and disease progression [87]. In this context, the reduction of CD68-positive microglia observed after riluzole treatment is consistent with a normalization of microglial phagocytic activity rather than its suppression.

Consistent with our findings, riluzole has been reported to reduce proinflammatory cytokine expression, promote M2-like polarization, and decrease CD68 levels in multiple disease models [53, 88, 89]. In addition, riluzole reverses the expression of inflammation-related gene networks implicated in AD pathology [39]. Together, these studies support the notion that riluzole biases microglial responses toward a neuroprotective and phagocytosis-competent state.

Morphological alterations further reflect microglial functional state. AD brains are characterized by an increased proportion of dystrophic (hypofunctional) microglia and a reduction in ramified surveillance microglia [31], consistent with our observations following Aβ oligomer exposure. Notably, riluzole reduced dystrophic microglia while increasing hypertrophic, injury-responsive microglia (Figure S10), suggesting restoration of an effective response to pathological stimuli.

At the structural level, Aβ-induced microglial activation is associated with shortened processes and reduced branching complexity [90]. In contrast, increased branching and territorial complexity correlate with enhanced Aβ uptake and clearance [75, 91]. The restoration of microglial branching complexity observed in our study therefore likely provides a morphological substrate that supports efficient amyloid handling.

Collectively, these findings indicate that riluzole preserves microglial recruitment while restoring functional and morphological features associated with effective Aβ clearance. This coordinated modulation of microglial state may contribute to reduced amyloid burden and limited synaptic loss.

## Conclusion

Using an Aβ oligomer–based injection model, we found that riluzole restores hippocampus-dependent memory not by suppressing glial recruitment, but by promoting coordinated functional and morphological remodelling of astrocytes and microglia towards neuroprotective phenotypes. Notably, these changes are accompanied by enhanced glial-mediated amyloid clearance, reduced complement-driven synaptic pruning, and preserved spine integrity. Taken together, these findings highlight coordinated glial remodeling as a physiologically relevant mechanism at a therapeutically actionable stage of AD. 

### Limitation

This study has several limitations that warrant consideration. A major limitation of this study is a 7-day intra-hippocampal cannula–based delivery of riluzole. Though it enables precise local delivery, it does not fully recapitulate clinically relevant systemic treatment regimen. Moreover, repeated intracerebral injections may itself influence local tissue responses within the target area. Secondly, multiple behavioral tests were conducted within a relatively short time window (1 week) which may introduce task-related carryover effects or stress-related influences on behavioral performance. Next, immunohistochemical analyses were performed after behavioral training such that observed changes may reflect combined effects of treatment and training. Importantly, although riluzole appeared to preserve synaptic structures, the functional competency of these recovered synapses was not directly assessed and requires electrophysiological validation. Finally, colocalization studies using conventional microscopy remain technically challenging; however, we are currently developing optimized protocols to address this limitation. Collectively, these considerations underscore the need for future studies incorporating clinically relevant systemic administration, temporally separated behavioral assessments, training-independent histological data, and functional evaluation of synaptic recovery.

## Supplementary Information

Supplementary Material 1. Video S1

Supplementary Material 2. Video S2

Supplementary Material 3.

## Data Availability

All data generated or analyzed during this study are included in this published article and its supplementary information files.

## Abbreviations

Aβ: Amyloid beta
AD: Alzheimer’s disease
ALS: Amyotrophic lateral sclerosis
APP: Amyloid precursor protein
AQP4: Aquaporin-4
C1q: Complement component 1q
C3: Complement component 3
CA1: Cornu ammonis 1
CD11b: Cluster of differentiation 11b
CD68: Cluster of differentiation 68
CR3: Complement receptor 3
E/I balance: Excitatory/inhibitory balance
I_NaP_: Persistent sodium current
LTP: Long-term potentiation
Nav1.6: Voltage-gated sodium channel 1.6
RLZ: Riluzole
TREM2: Triggering receptor expressed on myeloid cells 2
