# Supplementary material for: Riluzole shifts glial responses to protect synapses and memory in Aβ oligomer–treated rats
Source: J Neuroinflammation. 2026 Aug 25;23:323. doi: 10.1186/s12974-026-03972-3 (PMC13615615; doi:10.1186/s12974-026-03972-3)
Supplement: Supplementary file 1 [file 12974_2026_3972_MOESM3_ESM.docx]

**Supplementary Information**

**Title:**	Riluzole shifts glial responses to protect synapses and memory
in Aβ oligomer–treated rats

**Authors:**	Min-Kaung-Wint-Mon^1^, Hiroyuki Kida^1^, Ryoichi Kimura^2^, and Dai Mitsushima^1,3^

**Affiliations:** ^1^Department of Physiology, Yamaguchi University Graduate School of Medicine, Yamaguchi, 755-8505, Japan.

^2^Center for Liberal Arts and Sciences, Sanyo-Onoda City University, Sanyo-Onoda, Yamaguchi, 756-0884, Japan.

^3^The Research Institute for Time Studies, Yamaguchi University, Yamaguchi, 753-8511, Japan.

**Figure S1. Schematic timeline of the behavioral test battery.** Rats underwent the behavioral test battery after completion of riluzole or saline injection. It consisted of assessment of basic physiological functions (unboxed) and hippocampus‑dependent memory tasks (boxed). The sequence of the tests was optimized by choosing the least to the most stressful conditions for the rats, minimizing the interference among the tests and circumventing the influence of previous experience. Basic physiological tests were preceded by a 30‑minute interval, and memory tasks by a 2‑hour interval. ORT; object recognition task, OLT; object location task, OIPT; object-in-place task, OReT; object recency task, SRT; social recognition task, IA task; inhibitory avoidance task, FC test; fear conditioning test.

**Figure S2. Effect of riluzole on the engulfment of Aβ_1-42_ oligomers by astrocytes and microglia in the dorsal CA1 region.** Representative confocal super-resolution z-stack images showing the engulfment of Aβ_1-42_ oligomers by astrocytes (A) and microglia (B). The images were 3D-reconstructed using Imaris software (v.10.0.1; Bitplane). Surface rendering was applied to astrocytes and microglia, and internalized Aβ within the cells was visualized as cyan and unengulfed Aβ outside the cells as magenta using the masking tool. Scale bars: 5 μm.

**Figure S3. Riluzole does not alter basic locomotor, emotional, social, or sensory functions.** (A) Open-field test showing comparable total distance traveled, time spent in the center, latency to enter the center, center-entry frequency, and rearing activity across groups. (B) Total object exploration time was not affected by Aβ_1-42_ oligomers or riluzole treatment. (C) Social interaction time and social preference were similar among all groups. (D) Light/dark test showing no group differences in time spent in the lit compartment, latency to enter the light area, visit frequency, or rearing activity. (E) Flinch–jump test showing unaltered current thresholds for flinch, vocalization, and jump responses. Data are shown as individual values with mean ± SEM; sample sizes are indicated below each group.

**Figure S4. Effect of riluzole on Aβ recognition by CD11b in the dorsal CA1 region.** Representative images and quantification of colocalized Aβ/CD11b spots per mm². Colocalized Aβ/CD11b spots was increased in the Aβ+RLZ group. Arrowheads indicate colocalized puncta. The magnification is ×200. Scale bars: 40 μm. Sample sizes (number of animals) are shown below each bar. Data were presented as individual values with mean ± SEM. ***P* < 0.01 *vs* saline; ^##^*P* < 0.01 *vs* Aβ.

**Figure S5. Effect of riluzole on MAP2 expression in the dorsal CA1 region**. Representative images and quantification of MAP2 fluorescence intensity in the stratum radiatum of the dorsal CA1. The magnification is ×200. Scale bars: 100 μm. Sample sizes (number of animals) are shown below each bar. Data are presented as individual values with mean ± SEM. ***P* < 0.01 *vs* saline; ^##^*P* < 0.01 *vs* Aβ.

**Figure S6.** An overview of Golgi-stained CA1 neurons and representative dendritic spines across the CA1 sublayer**.** Scale bar: 100 μm with zoom-in 2 μm. SO; Stratum oriens, SR; Stratum radiatum, SLM; Stratum lacunosum-moleculare.

**Figure S7. Effects of riluzole on Aβ_1-42_ oligomer-induced synaptic loss of the dorsal CA1 region. (A)** Representative fluorescence images and semi-quantitative analysis of colocalized vGlut1/PSD95 puncta per 100 μm^2^ in the stratum lacunosum-moleculare of the dorsal region. The Aβ-induced reduction in synaptic puncta was reversed in the Aβ +RLZ group. **(B)** Quantitative analysis showed that Aβ oligomers did not significantly affect presynaptic vGlut1 puncta, but decreased total postsynaptic PSD95. This resulted in a decrease in colocalized synaptic puncta in the stratum radiatum and stratum lacunosum-moleculare of the dorsal CA1 region, respectively. Magnification is ×400 (digital zoom 3×), and the dotted circles indicate colocalized puncta. Scale bars: 5 μm. Sample sizes (number of animals) are shown below each bar. Data were presented as individual values with mean ± SEM. ** *p* < 0.01 *vs.* saline. ^#^ *p* < 0.05, ^##^ *p* < 0.01 *vs*. Aβ.

**Figure S8. Effects of riluzole on astrocytic marker expression in the dorsal CA1 region.** Representative fluorescence images of C3/GFAP⁺ (A) and S100A10/GFAP⁺ (B) cells. Arrowheads indicate double positive cells. Scale bars: 100 μm.

**Figure S9. Effects of riluzole on microglial marker expression in the dorsal CA1 region.** Representative fluorescence images of iNos/Iba1⁺ (A) and Arg1/Iba1⁺ (B) cells. Arrowheads indicate double positive cells. Scale bars: 100 μm.

**Figure S10. Effect of riluzole on Aβ_1-42_ oligomer-induced alteration in microglia**

**phenotype in the dorsal CA1 region.** Analysis of the morphological classification of microglia showed that the reduction of ramified microglia and the increase in dystrophic and rod-like microglia percentage induced by Aβ were reversed in the Aβ + RLZ group. The amoeboid type remained unchanged. Magnification is ×400 (digital zoom 3×). Scale bar: 20 μm. Sample sizes (number of animals) are shown below each bar. Data were presented as individual values with mean ± SEM. * *p* < 0.05; ** *p* < 0.01 *vs.* saline. ^#^ *p* < 0.05, ^##^ *p* < 0.01 *vs*. Aβ.

**Video S1.** (separate file) This video shows surface-rendered 3D reconstructions of an animal treated with Aβ and saline (see Figs 3B and S2). An example of lecanemab-positive astrocytes was shown.

**Video S2.** (separate file) This video shows surface-rendered 3D reconstructions of an animal treated with Aβ and RLZ (see Figs 3B and S2). An example of lecanemab-positive astrocytes was shown.

**Table S1**	Antibodies used in this study. The table summarizes information about supplier company, catalog number and concentration of use.

| Antibody | Dilution | Supplier | Catalog# | RRID |
| --- | --- | --- | --- | --- |
| Mouse anti-NeuN | 1:250 | Sigma-Aldrich | MAB377 | AB_2298772 |
| Mouse anti-MAP2 | 1:500 | Sigma-Aldrich | M9942 | AB_477256 |
| Mouse anti-C3 | 1:100 | Santa Cruz Biotechnology | SC-28294 | AB_627277 |
| Mouse anti-S100A10 | 1:100 | Cell Signaling Technology | 5529T | AB_10984111 |
| Mouse anti-CD11b | 1:200 | Sigma-Aldrich | CBL1512-25UG | AB_1136716 |
| Mouse anti-CD68 | 1:200 | Abcam | ab955 | AB_398071 |
| Mouse anti-iNos | 1:50 | Santa Cruz Biotechnology | SC-7271 | AB_2241060 |
| Mouse anti-arginase1 | 1:100 | Santa Cruz Biotechnology | SC-271430 | AB_10648473 |
| Mouse anti-PSD95 | 1:500 | Neuromab | 750-28 | AB_2292909 |
| Mouse anti-cathepsin D | 1:500 | Abcam | ab302649 | AB_2887401 |
| Rat anti-C1q | 1:500 | Santa Cruz Biotechnology | SC-58920 | AB_627885 |
| Rabbit anti-NeuN | 1:250 | Abcam | ab177487 | AB_2532109 |
| Rabbit anti-cleaved caspase 3 | 1:100 | Cell Signaling Technology | 9664T | AB_2070042 |
| Rabbit anti-PSD95 | 1:500 | Abcam | ab18258 | AB_444362 |
| Rabbit anti-GFAP | 1:1000 | Dako | Z0334 | AB_10013382 |
| Rabbit anti-Iba1 | 1:250 | Wako | 019-19741 | AB_839504 |
| Guinea pig anti-vGlut1 | 1:1000 | Wako | 013-2811 | AB_2301751 |
| Lecanemab | 1:500 | Selleck | A3112 | - |
| Goat anti-mouse Alexa 594 | 1:500 | Abcam | ab150116 | AB_2650601 |
| Goat anti-mouse FITC | 1:500 | Jackson ImmunoResearch | F-2761 | AB_2536524 |
| Goat anti-mouse 647 | 1:500 | Abcam | ab150115 | AB_2921069 |
| Goat anti-rabbit Texas Red | 1:500 | Jackson ImmunoResearch | 72626 | AB_2338001 |
| Goat anti-rabbit FITC | 1:500 | Chemicon | AP307F | AB_92652 |
| Goat anti- guinea pig Alexa 488 | 1:500 | Abcam | ab150185 | AB_2736871 |
| Goat anti-human Alexa 488 | 1:500 | Invitrogen | A56869 | AB_2937242 |

**Table S2**	Summary of statistical results from **Figure 4**

| Parameter | ANOVA | Bonferroni’s test |
| --- | --- | --- |
| **Figure 4C** | | |
| Total spines  (per 20 µm) | Aβ: *F*_1, 12_ = 28.060, *p* = 0.0002  Riluzole: *F*_1, 12_ = 23.863, *p* = 0.0004  Interaction: *F*_1, 12_ = 22.280, *p* = 0.0005 | Aβ *vs*. saline, *p* < 0.0001  Aβ *vs*. Aβ+RLZ, *p* = 0.0001 |
| Thin spines | Aβ: *F*_1, 12_ = 1.597, *p* = 0.2303  Riluzole: *F*_1, 12_ = 4.480, *p* = 0.0559  Interaction: *F*_1, 12_ = 1.597, *p* = 0.2303 | Aβ *vs*. saline, *p* = 0.5949  Aβ *vs*. Aβ+RLZ, *p* = 0.2047 |
| Mushroom spines | Aβ: *F*_1, 12_ = 36.988, *p* < 0.0001  Riluzole: *F*_1, 12_ = 23.591, *p* = 0.0004  Interaction: *F*_1, 12_ = 18.018, *p* = 0.0011 | Aβ *vs*. saline, *p* < 0.0001  Aβ *vs*. Aβ+RLZ, *p* = 0.0002 |
| Stubby spines | Aβ: *F*_1, 12_ = 19.832, *p* = 0.0008  Riluzole: *F*_1, 12_ = 11.833, *p* = 0.0049  Interaction: *F*_1, 12_ = 16.830, *p* = 0.0015 | Aβ *vs*. saline, *p* = 0.0003  Aβ *vs*. Aβ+RLZ, *p* = 0.0011 |
| **Figure 4D** | | |
| Head area (µm^2^) | Aβ: *F*_1, 428_ = 8.600, *p* = 0.0035  Riluzole: *F*_1, 428_ = 2.820, *p* = 0.0938  Interaction: *F*_1, 428_ = 5.786, *p* = 0.0166 | Aβ *vs*. saline, *p* = 0.0010  Aβ *vs*. Aβ+RLZ, *p* = 0.0224 |
| Neck length (µm) | Aβ: *F*_1, 428_ = 1.898, *p* = 0.1690  Riluzole: *F*_1, 428_ = 7.642, *p* = 0.0059  Interaction: *F*_1, 430_ = 8.125, *p* = 0.0046 | Aβ *vs*. saline, *p* = 0.0165  Aβ *vs*. Aβ+RLZ, *p* = 0.0004 |
| Neck width (µm) | Aβ: *F*_1, 428_ =, *p* < 0.0001  Riluzole: *F*_1, 428_ = 67.004, *p* < 0.0001  Interaction: *F*_1, 428_ = 75.174, *p* < 0.0001 | Aβ *vs*. saline, *p* = 0.7588  Aβ *vs*. Aβ+RLZ, *p* = 0.2832 |
| **Figure 4E** | | |
| Total spines  (per 20 µm) | Aβ: *F*_1, 12_ = 28.060, *p* = 0.0002  Riluzole: *F*_1, 12_ = 23.863, *p* = 0.0004  Interaction: *F*_1, 12_ = 22.279, *p* = 0.0005 | Aβ *vs*. saline, *p* < 0.0001  Aβ *vs*. Aβ+RLZ, *p* = 0.0001 |
| Thin spines | Aβ: *F*_1, 12_ = 17.667, *p* = 0.0012  Riluzole: *F*_1, 12_ = 3.689, *p* = 0.0788  Interaction: *F*_1, 12_ = 9.547, *p* = 0.0094 | Aβ *vs*. saline, *p* = 0.0014  Aβ *vs*. Aβ+RLZ, *p* = 0.0243 |
| Mushroom spines | Aβ: *F*_1, 12_ = 4.374, *p* = 0.0584  Riluzole: *F*_1, 12_ = 9.289, *p* = 0.0101  Interaction: *F*_1, 12_ = 4.196, *p* = 0.0631 | Aβ *vs*. saline, *p* = 0.0760  Aβ *vs*. Aβ+RLZ, *p* = 0.0217 |
| Stubby spines | Aβ: *F*_1, 12_ = 10.836, *p* = 0.0064  Riluzole: *F*_1, 12_ = 5.107, *p* = 0.0432  Interaction: *F*_1, 12_ = 10.616, *p* = 0.0069 | Aβ *vs*. saline, *p* = 0.0035  Aβ *vs*. Aβ+RLZ, *p* = 0.0126 |
| **Figure 4F** | | |
| Head area (µm^2^) | Aβ: *F*_1, 422_ = 40.479, *p* < 0.0001  Riluzole: *F*_1, 422_ = 14.006, *p* = 0.0002  Interaction: *F*_1, 422_ = 25.900, *p* < 0.0001 | Aβ *vs*. saline, *p* < 0.0001  Aβ *vs*. Aβ+RLZ, *p* < 0.0001 |
| Neck length (µm) | Aβ: *F*_1, 422_ = 6.772, *p* = 0.0096  Riluzole: *F*_1, 422_ = 2.837, *p* = 0.0928  Interaction: *F*_1, 422_ = 4.646, *p* = 0.0317 | Aβ *vs*. saline, *p* = 0.0046  Aβ *vs*. Aβ+RLZ, *p* = 0.0438 |
| Neck width (µm) | Aβ: *F*_1, 422_ =45.320, *p* < 0.0001  Riluzole: *F*_1, 422_ = 12.037, *p* = 0.0006  Interaction: *F*_1, 422_ = 25.244, *p* < 0.0001 | Aβ *vs*. saline, *p* < 0.0001  Aβ *vs*. Aβ+RLZ, *p* < 0.0001 |
| **Figure 4G** | | |
| Total spines  (per 20 µm) | Aβ: *F*_1, 12_ = 9.693, *p* = 0.0090  Riluzole: *F*_1, 12_ = 9.008, *p* = 0.0108  Interaction: *F*_1, 12_ = 9.313, *p* = 0.0101 | Aβ *vs*. saline, *p* = 0.0056  Aβ *vs*. Aβ+RLZ, *p* = 0.0063 |
| Thin spines | Aβ: *F*_1, 12_ = 5.109, *p* = 0.0432  Riluzole: *F*_1, 12_ = 4.323, *p* = 0.0597  Interaction: *F*_1, 12_ = 4.974, *p* = 0.0456 | Aβ *vs*. saline, *p* = 0.0479  Aβ *vs*. Aβ+RLZ, *p* = 0.0608 |
| Mushroom spines | Aβ: *F*_1, 12_ = 9.957, *p* = 0.0083  Riluzole: *F*_1, 12_ = 3.585, *p* = 0.0827  Interaction: *F*_1, 12_ = 10.139, *p* = 0.0079 | Aβ *vs*. saline, *p* = 0.0045  Aβ *vs*. Aβ+RLZ, *p* = 0.0223 |
| Stubby spines | Aβ: *F*_1, 12_ = 0.010, *p* = 0.9239  Riluzole: *F*_1, 12_ = 1.664, *p* = 0.2213  Interaction: *F*_1, 12_ = 0.010, *p* = 0.9239= | Aβ *vs*. saline, *p* > 0.9999  Aβ *vs*. Aβ+RLZ, *p* > 0.9999 |
| **Figure 4H** | | |
| Head area (µm^2^) | Aβ: *F*_1, 407_ = 7.877, *p* = 0.0052  Riluzole: *F*_1, 407_ = 4.809, *p* = 0.0289  Interaction: *F*_1, 407_ = 2.564, *p* = 0.1101 | Aβ *vs*. saline, *p* = 0.0110  Aβ *vs*. Aβ+RLZ, *p* = 0.0434 |
| Neck length (µm) | Aβ: *F*_1, 407_ = 2.290, *p* = 0.1310  Riluzole: *F*_1, 407_ = 7.178, *p* = 0.0077  Interaction: *F*_1, 407_ = 9.573, *p* < 0.0021 | Aβ *vs*. saline, *p* = 0.0068  Aβ *vs*. Aβ+RLZ, *p* = 0.0003 |
| Neck width (µm) | Aβ: *F*_1, 407_ =18.177, *p* < 0.0001  Riluzole: *F*_1, 407_ = 15.166, *p* = 0.0001  Interaction: *F*_1, 407_ = 8.049, *p* = 0.0048 | Aβ *vs*. saline, *p* < 0.0001  Aβ *vs*. Aβ+RLZ, *p* < 0.0001 |

**Table S3**	Summary of statistical results from **Figure S**7

| Parameter | ANOVA | Bonferroni’s test |
| --- | --- | --- |
| **Figure S7A** | | |
| Colocalized puncta per 100 µm^2^ | Aβ: *F*_1, 13_ = 14.809, *p* = 0.0020  Riluzole: *F*_1, 13_ = 9.068, *p* = 0.0100  Interaction: *F*_1, 13_ = 9.495, *p* = 0.0088 | Aβ *vs*. saline, *p* = 0.0014  Aβ *vs*. Aβ+RLZ, *p* = 0.0041 |
| **Figure S7B** | | |
| vGlut1^+^ puncta (CA1-SR) | Aβ: *F*_1, 13_ = 1.851, *p* = 0.8921  Riluzole: *F*_1, 13_ = 0.0020, *p* = 0.9653  Interaction: *F*_1, 13_ = 0.019, *p* = 0.8921 | Aβ *vs*. saline, *p* > 0.9999  Aβ *vs*. Aβ+RLZ, *p* > 0.9999 |
| PSD95^+^ puncta (CA1-SR) | Aβ: *F*_1, 13_ = 7.185, *p* = 0.189  Riluzole: *F*_1, 13_ = 1.824, *p* = 0.2000  Interaction: *F*_1, 13_ = 9.317, *p* = 0.0093 | Aβ *vs*. saline, *p* = 0.0067  Aβ *vs*. Aβ+RLZ, *p* = 0.0419 |
| vGlut1^+^ puncta (CA1-SLM) | Aβ: *F*_1, 13_ = 3.577, *p* = 0.0811  Riluzole: *F*_1, 13_ = 1.527, *p* = 0.2384  Interaction: *F*_1, 13_ = 3.455, *p* = 0.0858 | Aβ *vs*. saline, *p* = 0.1042  Aβ *vs*. Aβ+RLZ, *p* = 0.2553 |
| PSD95^+^ puncta (CA1-SLM) | Aβ: *F*_1, 13_ = 5.315, *p* = 0.0383  Riluzole: *F*_1, 13_ = 4.371, *p* = 0.0568  Interaction: *F*_1, 13_ = 6.994, *p* = 0.0202 | Aβ *vs*. saline, *p* = 0.0195  Aβ *vs*. Aβ+RLZ, *p* = 0.0263 |

**Table S4**	Summary of statistical results from **Figure S10**

| Parameter | ANOVA | Bonferroni’s test |
| --- | --- | --- |
| Ramified | Aβ: *F*_1, 13_ = 72.080, *p* < 0.0001  Riluzole: *F*_1, 13_ = 37.568, *p* < 0.0001  Interaction: *F*_1, 13_ = 21.198, *p* = 0.0005 | Aβ *vs*. saline, *p* < 0.0001  Aβ *vs*. Aβ+RLZ, *p* < 0.0001 |
| Hypertrophic | Aβ: *F*_1, 13_ = 0.127, *p* = 0.7271  Riluzole: *F*_1, 13_ = 2.952, *p* = 0.1095  Interaction: *F*_1, 13_ = 7.245, *p* = 0.0185 | Aβ *vs*. saline, *p* = 0.6816  Aβ *vs*. Aβ+RLZ, *p* = 0.0415 |
| Dystrophic | Aβ: *F*_1, 13_ = 250.946, *p* < 0.0001  Riluzole: *F*_1, 13_ = 176.087, *p* < 0.0001  Interaction: *F*_1, 13_= 219.821, *p* < 0.0001 | Aβ *vs*. saline, *p* < 0.0001  Aβ *vs*. Aβ+RLZ, *p* < 0.0001 |
| Amoeboid | Aβ: *F_1_*_, 13_ = 26.159, *p* = 0.0002  Riluzole: *F*_1, 13_ = 0.095, *p* = 0.7628  Interaction: *F*_1, 13_ = 1.648, *p* = 0.2217 | Aβ *vs*. saline, *p* = 0.0931  saline *vs*. Aβ+RLZ, *p* = 0.0147 |
| Rod-like | Aβ: *F*_1, 13_ = 14.969, *p* = 0.0019  Riluzole: *F*_1, 13_ = 4.917, *p* = 0.0450  Interaction: *F*_1, 13_ = 6.020, *p* = 0.0290 | Aβ *vs*. saline, *p* = 0.0030  Aβ *vs*. Aβ+RLZ, *p* = 0.0288 |
